# Tb^3+^‐Doped Glass‐Ceramic Scintillating Plates and Fibers for X‐Ray Imaging and Flexible Detection

**DOI:** 10.1002/advs.74568

**Published:** 2026-02-25

**Authors:** Songxuan Liu, Ping Zhang, Panpan Li, Yao Ji, Zhiguo Xia, Weichao Wang, Qinyuan Zhang

**Affiliations:** ^1^ State Key Laboratory of Luminescent Materials and Devices Guangdong Provincial Key Laboratory of Fiber Laser Materials and Applied Techniques School of Physics and Optoelectronics South China University of Technology Guangzhou P. R. China

**Keywords:** fiber‐optic sensing, flexible detection, glass‐ceramic scintillator, X‐ray imaging

## Abstract

The development of flexible and wearable radiation detection technology is urgently needed for medical imaging, non‐destructive testing, and personal dosimetry. Although bulk single‐crystal and ceramic scintillators suffer from intrinsic brittleness and scalability issues, glass scintillators typically show limited light yield. To overcome these challenges, we have developed a Tb^3+^‐doped oxyfluoride glass‐ceramic scintillator via an integrated approach combining phase‐diagram guidance and molecular dynamics simulations. Based on the controlled precipitation of Ba_2_GdF_7_ nanocrystals, the as‐prepared glass‐ceramic scintillator achieves a light yield of 41 800 photons/MeV (418% of BGO). It also achieves a high X‐ray imaging spatial resolution of 25.3 lp/mm with large‐area scintillating plates and demonstrates exceptional thermal quenching resistance. Leveraging the superior processability of the glass matrix, we further fabricated a flexible scintillation fiber for remote, highly sensitive radiation dosimetry. The fiber sensor exhibits a sensitivity of 224 nGy/s, stability over 240 on‐off irradiation cycles, and excellent linearity (R^2^ = 0.9999). This work not only presents a robust glass‐ceramic scintillator material design strategy but also demonstrates its potential in next‐generation distributed radiation detection systems using large‐area scintillating plates and flexible glass fibers.

## Graphical abstract

A Tb^3+^‐doped glass‐ceramic scintillator, with controlled Ba_2_GdF_7_ nanocrystal precipitation, was developed through a rational design strategy guided by phase diagrams and molecular dynamics simulations. The obtained material exhibits a high light yield of 41 800 photons/MeV (418%/BGO), a spatial resolution of 25.3 lp/mm, and exceptional thermal stability, enabling high‐resolution X‐ray imaging for non‐destructive testing applications.

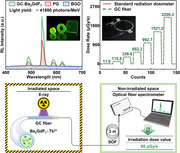

## 1 Introduction

Growing demands for global public health monitoring and advanced medical diagnostics have highlighted the potential of flexible electronics and wearable devices in medical imaging, non‐destructive testing, and personal radiation dosimetry. This creates an urgent need for high‐performance scintillator materials that offer versatile processability [1, 2, 3, 4]. As core components that convert high‐energy radiation (X‐rays or γ‐rays) into ultraviolet or visible photons, scintillators govern the detection efficiency of related systems. An ideal scintillator should feature a high absorption coefficient, superior scintillation properties (high light yield, fast decay), excellent stability, and compatibility with flexible device architectures [5, 6, 7]. However, conventional high‐performance scintillators, such as Bi_4_Ge_3_O_12_ (BGO), LuAl_5_O_12_: Ce (LuAG: Ce), CsI: Tl, and Gd_2_Al_2_Ga_3_O_12_ (GAGG) single crystals or ceramics [8], suffer from demanding growth conditions, high cost, inherent brittleness, and processing difficulties, which severely restrict their applications in flexible or complex‐shaped devices [9, 10]. Although emerging scintillators (e.g., perovskite quantum dots and polymer semiconductors) [11, 12, 13, 14] have achieved progress in light output, their commercialization is hampered by toxicity or insufficient operational stability. Beyond performance enhancement, the current core scientific challenge also involves understanding and mitigating degradation mechanisms (e.g., ion migration and photo‐oxidation) that limit device longevity. Addressing this stability issue requires coordinated research focusing on material encapsulation, interface engineering, and innovative compositional design.

In this context, oxide glass scintillators have attracted considerable attention due to their facile fabrication, low cost, and excellent formability, making them particularly suitable for large‐scale panels and optical fibers [15, 16]. Among various luminescent centers, Tb^3+^ stands out for its strong green emission at ∼ 540 nm, which matches the spectral response of silicon‐based detectors [17]. However, the performance of Tb^3+^‐doped glass scintillators is often limited by the high phonon energy of the oxide matrix, which causes severe non‐radiative relaxation and low luminescence efficiency [18]. Transparent glass‐ceramics, incorporating functional nanocrystals in a residual glass matrix, offer an effective strategy to address this limitation [19, 20]. This unique composite structure leverages the favorable luminescent environment of the crystalline phase (e.g., low phonon energy) while retaining the excellent mechanical strength and processability of glass. Fluoride crystals are particularly desirable for this purpose. Although several fluoride‐based glass‐ceramic scintillators (e.g., Sr_2_GdF_7_ [21], Na_5_Gd_9_F_32_ [22], BaGdF_5_ [23], and KTbGdF_10_ [24]) have been developed, they still require improvement in terms of light yield or thermal stability, largely due to empirical composition design and insufficient microstructure control. To enhance scintillator performance, introducing elements with high atomic numbers into the glass composition represents a direct and effective approach (e.g., Ba: Z = 56, Gd: Z = 64). Ba ions provide strong X‐ray stopping power [25], while Gd ions serve as efficient sensitizers, absorbing X‐ray energy and transferring it to activators such as Tb^3+^ [26]. Thus, controlled precipitation of Ba─Gd─F crystalline phases represents a promising route toward high‐performance scintillators.

Herein, we report a rationally designed Tb^3+^‐doped oxyfluoride glass‐ceramic scintillator with significantly enhanced performance achieved by controlled precipitation of Ba_2_GdF_7_ nanocrystals. Our material design strategy, guided by phase diagram thermodynamics and molecular dynamics (MD) simulations, enables optimized nanocrystallization, yielding a high light output of 41 800 photons/MeV (418% of BGO) and exceptional thermal stability. Beyond a high‐resolution X‐ray imaging panel with excellent scintillation performance, we further fabricated a flexible fiber scintillator specifically engineered for remote and highly sensitive radiation dosimetry. This fiber device directly meets the growing demand for adaptable, personal, and distributed radiation monitoring. Our study not only delivers a high‐performance scintillator but also establishes a generalizable design framework for functional materials that balance superior performance and excellent processability.

## 2 Results and Discussion

### 2.1 Material Design

**FIGURE 1:**
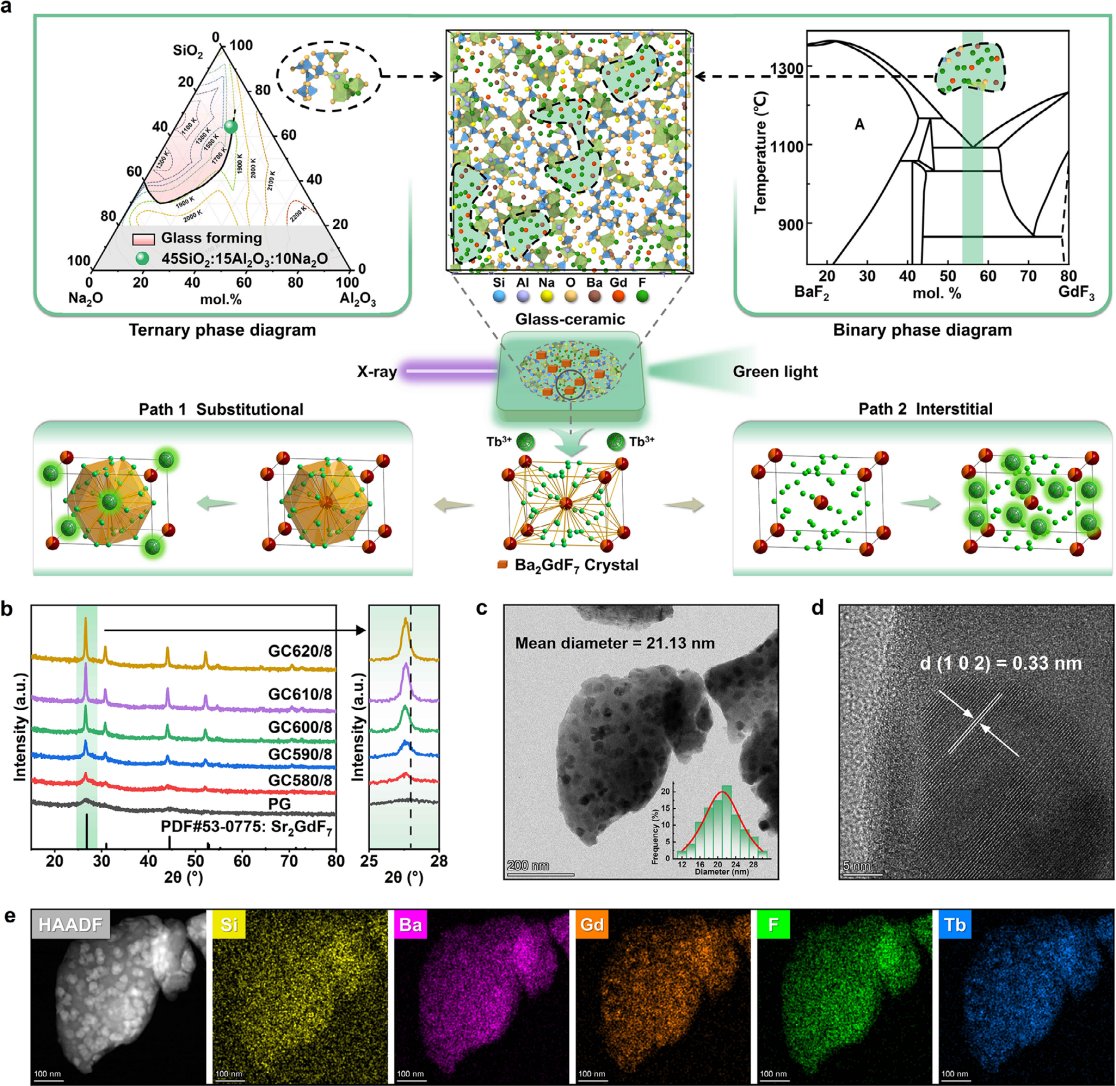
(a) The complete design process of glass‐ceramic materials, from phase diagram screening and MD simulation for structural prediction to the Tb^3+^ doping mechanism; (b) XRD patterns of the precursor glass and glass‐ceramics heat‐treated at different temperatures; (c) TEM image and grain size distribution histogram, (d) HRTEM image, and (e) EDS elemental mapping of the glass‐ceramic.

The high atomic numbers of Ba (Z = 56) and Gd (Z = 64) enable strong X‐ray absorption capabilities. Efficient conversion of X‐ray photon energy to secondary electrons is key to a high signal‐to‐noise ratio in imaging. Gd^3+^ ions play a dual role: improving X‐ray absorption and sensitizing resonance energy transfer to Tb^3+^ luminescent centers. This dual functionality maximizes absorbed X‐ray energy utilization and reduces non‐radiative losses. From a materials design perspective, fluoride crystal precipitation in the glass matrix reduces phonon energy, benefiting activator ion luminescence. The Ba─Gd─F crystalline phase is advantageous, enabling Ba^2+^/Gd^3+^ co‐incorporation and synergistic effects [27]. The crystal field provides a more symmetrical coordination sphere for dopant ions, enhancing radiative transition probabilities. Thus, controlling Ba─Gd─F phase crystallization to optimize Tb^3+^ local coordination is a practically valuable research direction. We focused on controlled crystallization and optical transparency (critical for scintillation), selecting a SiO_2_‐Al_2_O_3_‐Na_2_O silicate glass system with BaF_2_ and GdF_3_ (≥ 99.99%) serving as crystal precursors. This base glass composition enables controlled phase separation (a crystallization precursor) and favorable optical properties. Based on the ternary phase diagram (Figure 1a) [28, 29], we selected the composition with a high aluminosilicate crystallization temperature (*T_x_* > 750 C) to widen the fluoride crystallization processing window. Near the BaF_2_‐GdF_3_ eutectic point (2:1) [30, 31], we chose a composition to lower crystallization temperature and promote single Ba_2_GdF_7_ phase formation (Figure 1a). This ensures homogeneous nanocrystal distribution and preserves optical transparency.

MD simulations revealed the glass network's structural features (Figure 1a) and atomic‐level crystallization mechanisms. Under simulated rapid cooling, a clear phase separation occurred between an aluminosilicate network ([SiO_4_] and [AlO_4_] tetrahedra) and a fluorine‐rich region (dashed shading). The simulated cooling rate (5 K/ps) is much higher than the experimental one, but MD simulations remain valuable for revealing local coordination and phase separation trends to guide design, even if the precise nanostructure differs. This nanoscale phase separation is fundamental to controlled crystallization, as fluorine‐rich regions serve as nucleation sites for Ba_2_GdF_7_ crystal formation. Fluorine (F) plays a dual role: forming Al(O, F)_x_ polyhedral units that stabilize network structure, and also associating with modifier ions, enhancing mobility for crystal nucleation and growth. Radial distribution function (RDF) analysis (Figure S1) confirmed that oxygen (O) primarily bonds with silicon (Si) to form a silicate framework, while fluorine (F) predominantly coordinates with gadolinium (Gd), aluminum (Al), sodium (Na), and barium (Ba), forming fluorine‐rich phases conducive to Ba─Gd─F crystals [32]. This preferential coordination explains fluoride phase formation (over other phases) and provides a theoretical basis for targeted Ba_2_GdF_7_ precipitation.

Figure 1a illustrates Tb^3+^ ion doping mechanisms in the Ba_2_GdF_7_ crystalline phase [33, 34]. Tb^3+^ ions incorporate into the crystal lattice through two primary pathways: i) substitutional doping, in which Tb^3+^ replaces Gd^3+^ at lattice sites, and ii) interstitial doping, where Tb^3+^ occupies interstitial positions. Substitutional doping is particularly favorable due to the similar ionic radii and charge states of Gd^3+^ and Tb^3+^, minimizing lattice distortion and enabling efficient activator incorporation. The fluoride crystalline phase offers a favorable luminescent environment for Tb^3+^ radiative transitions, with low phonon energy suppressing non‐radiative decay. More importantly, the ordered structure of Ba_2_GdF_7_ boosts energy transfer efficiency from Gd^3+^ to Tb^3+^. Shortened sensitizer‐activator distances strengthen dipole‐dipole interactions, improving energy transfer probability. Experimental results confirm that Ba_2_GdF_7_ nanocrystal precipitation optimizes Tb^3+^ local coordination and enhances luminescence via improved energy transfer. This design (integrating thermodynamic guidance and atomic‐level structural insights) underpins advanced scintillation materials with tailored properties.

### 2.2 Composition Optimization of Glass‐Ceramics

To identify the optimal crystallization composition, a series of precursor glasses with graded BaF_2_/GdF_3_ ratios was prepared via the melt‐quenching method. The base composition was 45SiO_2_‐15Al_2_O_3_‐10Na_2_O‐(30‐*x*)BaF_2_‐*x*GdF_3_ (*x* = 8, 10, 12 mol%; denoted as Ba/Gd = 22:8, 20:10, 18:12). This systematic variation enables a precise investigation into how Gd^3+^ content influences structural evolution and crystallization behavior, which is crucial for understanding composition‐structure‐property relationships in this complex system. MD simulations were first used to predict and compare the local structures of these three compositions. The radial distribution functions (RDFs) of Ba─F, Gd─F, and F─F pairs (Figure S2) exhibited non‐monotonic variations with increasing GdF_3_ content, suggesting an optimal ratio for fluorine‐rich cluster formation. Notably, the Ba/Gd = 20:10 composition exhibited the highest coordination numbers for these pairs, indicating a structure more conducive to forming a percolating fluorine‐rich environment for subsequent crystallization. This optimal connectivity between modifier cations and F^−^ ions lowers the nucleation energy barrier and promotes homogeneous crystallization in the glass matrix. Further RDF analysis for Na─F and Na─O pairs (Figure S3) provided insights into the phase separation mechanism. In the Ba/Gd = 20:10 glass, Na^+^ ions preferentially partitioned into the oxygen‐rich silicate network instead of associating with F^−^ ions. This preferential distribution promotes clear phase separation between the silicate network and fluoride‐rich regions, effectively guiding Ba─Gd─F crystal precipitation by minimizing competitive interactions that would otherwise hinder crystallization. Bond angle distribution analysis (Figure S4a) showed that although the O─Si─O angle was centered at ∼ 108° (characteristic of tetrahedral coordination), its distribution broadened markedly for the Ba/Gd = 20:10 composition. This enhanced short‐range disorder suggests increased structural flexibility around silicate units, likely induced by the incipient fluoride crystallization that creates strain fields in the glass network. The F─Ba─F and F─Gd─F bond angles (Figure S4b,c) were centered at ∼60° and ∼120°, reflecting complex coordination geometries matching previously reported crystalline phases [35]. This angular distribution provides preliminary evidence for pre‐nucleation clusters adopting configurations similar to the final crystalline structure.

Experimental validation through X‐ray diffraction (XRD) analysis of glass samples heat‐treated at 660 C for 2 h (Figure S5) confirmed the computational predictions. All compositions showed clear crystallization peaks, but the Ba/Gd = 20:10 sample exhibited the highest diffraction intensity, indicating superior crystallization tendency and potentially higher crystallinity. Although no standard reference pattern (PDF card) is available for Ba_2_GdF_7_, its diffraction pattern closely matched that of Sr_2_GdF_7_ (PDF#53‐0775), with all peaks systematically shifted to lower angles (inset of Figure S5). This shift is consistent with the larger ionic radius of Ba^2+^ (1.42 Å) than Sr^2+^ (1.26 Å), which increases the interplanar spacing (d), as predicted by Bragg's law [36]. The results confirm the successful precipitation of the Ba_2_GdF_7_ crystals, with the optimal composition yielding the most pronounced crystallization.

With the optimal base composition (Ba/Gd = 20:10) for Ba_2_GdF_7_ crystallization identified, Tb^3+^ doping levels were systematically varied to maximize scintillation performance. Glasses of 45SiO_2_‐15Al_2_O_3_‐10Na_2_O‐20BaF_2_‐10GdF_3_‐yTbF_3_ (y = 1, 2, 4, 6, 8, 10, 12, 14, and 16 mol%) were prepared, denoted as 1Tb‐16Tb. Visual inspection under sunlight and UV light (Figure S6a) showed high transparency across all samples, indicating that Tb^3+^ doping, even at high concentrations, did not compromise the optical quality of the glass‐ceramics, a critical requirement for scintillation applications. Photoluminescence spectra (PL) under 377 nm excitation (Figure S6b) exhibited characteristic Tb^3+^ emissions at 490 nm (^5^D_4_→^7^F_6_), 543 nm (^5^D_4_→^7^F_5_), 585 nm (^5^D_4_→^7^F_4_), and 622 nm (^5^D_4_→^7^F_3_), with the 543 nm green emission being the most intense due to its electric dipole nature. To assess scintillation performance, radioluminescence (RL) properties under X‐ray irradiation were measured (Figure S6c). The light yield, calculated from the integrated X‐ray‐excited emission (Figure S6d), trended with PL intensity, both peaking at y = 12 mol% (12Tb). Significant quenching is induced by Tb^3+^ ion aggregation and cross‐relaxation at concentrations above 12 mol%, which further enhances non‐radiative relaxation and impairs luminescence performance. This PL‐RL concordance confirms that energy transfer from the host to Tb^3+^ ions operates consistently under different excitation sources, indicating that 12 mol% is the optimal Tb^3+^ concentration before concentration quenching occurs. This optimal doping level was consequently adopted for all subsequent investigations to ensure maximum scintillation performance.

### 2.3 Controlled Crystallization of Glass‐Ceramics

To achieve glass‐ceramics with optimal microstructure and scintillation performance, the thermal behavior of the 12Tb precursor glass was characterized. Differential scanning calorimetry (DSC) analysis at various heating rates (15–30 C/min, Figure S7) revealed a glass transition temperature at ∼550 C and two crystallization exotherms at ∼590 C and ∼810°C, corresponding to fluoride and silicate phase precipitation, respectively. The large temperature gap (ΔT ≈ 220°C) between the two crystallization peaks provides a wide processing window, allowing selective crystallization of the target Ba_2_GdF_7_ phase without interference from the silicate phase. This is key for microstructure regulation. Via the Kissinger equation [37] (Figure S8a), the crystallization activation energy of Ba_2_GdF_7_ was calculated as 166.7 ± 9 kJ/mol, much lower than that of the silicate phase (Figure S8b). This lower energy barrier confirms the preferential crystallization of the fluoride phase and explains the observed crystallization sequence from a kinetic perspective. Guided by these thermal parameters, isothermal heat treatments were performed at 580, 590, 600, 610, and 620 C for 8 h (samples GC580/8 to GC620/8) to investigate crystallization evolution systematically. XRD patterns (Figure 1b) confirmed the exclusive precipitation of Ba_2_GdF_7_ in all samples with no detectable secondary phases, demonstrating high phase selectivity achieved via the composition design. Increasing annealing temperature enhanced crystallinity (evidenced by elevated peak intensities) while peak widths remained unchanged, indicating nanoscale crystallites throughout the temperature range. Scherrer analysis estimated crystallite sizes in the range of 15–30 nm, with the optimal GC610/8 sample at ∼21 nm. This nanocrystalline dimension is vital for scintillation applications as it minimizes light scattering losses while providing a sufficient crystal field to enhance luminescence efficiency. Microstructure analysis by transmission electron microscopy (TEM) revealed spherical nanocrystals uniformly dispersed in the glass matrix (Figure 1c), ensuring high optical transparency and efficient energy transfer. High‐resolution TEM (HRTEM) images (Figure 1d) showed clear lattice fringes with a d‐spacing of 0.33 nm, matching the (102) plane of the isostructural Sr_2_GdF_7_. Well‐defined lattice fringes and clear crystallographic orientation across multiple nanocrystals indicate high crystalline quality despite their nanoscale dimensions. Energy‐dispersive X‐ray spectroscopy (EDS) mapping (Figure 1e; Figure S9) confirmed pronounced enrichment of Ba, Gd, Tb, and F in nanocrystals and their depletion in the glass matrix. This elemental partitioning confirms effective Tb^3+^ incorporation into the crystalline phase, which is crucial for achieving the desired luminescence properties. Consistent with Rayleigh scattering theory, increased heat treatment temperature led to larger crystallites and higher volume fraction, resulting in decreased UV transparency while maintaining high visible‐light transmittance (Figure S10). This optical behavior is characteristic of well‐controlled nanocrystallization, where the crystal size remains below the critical scattering threshold for visible wavelengths. The correlation between optical properties and visual appearance under daylight and UV illumination (Figure S11) further confirms the successful preparation of transparent glass‐ceramics with controlled nanocrystallization. This work demonstrates that coupling thermal analysis with precise processing conditions is critical for tailoring microstructure‐property relationships in functional glass‐ceramics.

### 2.4 Radioluminescence Properties of Glass‐Ceramic Scintillators

Glass‐ceramics containing Ba_2_GdF_7_ nanocrystals with varying crystallization degrees were prepared via controlled heat treatment, enabling systematic investigation of the structure‐property relationship. The photoluminescence excitation (monitored at 543 nm) and emission (excited at 377 nm) spectra of the precursor glass and glass‐ceramics are shown in Figure S12a,c. The excitation spectra had characteristic peaks of Gd^3+^ (273 nm) and Tb^3+^ (377 nm), with the latter being the most intense, indicating efficient direct excitation of Tb^3+^ ions. Under 377 nm excitation, all samples showed typical Tb^3+^ emission bands centered at 490 nm (^5^D_4_→^7^F_6_), 543 nm (^5^D_4_→^7^F_5_), 585 nm (^5^D_4_→^7^F_4_), and 622 nm (^5^D_4_→^7^F_3_), with the 543 nm green emission dominating due to its hypersensitivity nature. The emission intensity increased with heat treatment temperature up to 610 C (GC610/8), demonstrating that the Ba_2_GdF_7_ crystallization improves the luminescence environment of Tb^3+^ ions by providing a lower phonon energy matrix and a more symmetrical crystal field. The enhancement is attributed to the increased partitioning of Tb^3+^ ions into the crystalline phase, suppressing non‐radiative relaxation. Beyond 610 C, the intensity decrease is likely due to rare‐earth ion clustering within the nanocrystals and reduced energy transfer efficiency from excessive crystal growth, which promotes cross‐relaxation between Tb^3+^ ions. Fluorescence decay curves of the 543 nm emission (λ_ex_ = 377 nm) are shown in Figure S12e, with lifetimes summarized in Table S1. The gradual decrease in Tb^3+^ lifetime from 2.555 ms (precursor glass) to 2.211 ms (GC620/8) reflects changes in the local environment around Tb^3+^. The shorter lifetimes in glass‐ceramics suggest enhanced radiative transition probabilities in the crystalline environment, consistent with the observed intensity enhancement. Under 273 nm excitation (Figure S12b), emissions from Gd^3+^ and Tb^3+^ were observed. However, Tb^3+^ emission was stronger under 377 nm direct excitation (Figure S12d), showing that direct Tb^3+^ excitation is more efficient than Gd^3+^→Tb^3+^ energy transfer. Remarkably, the undoped glass (0Tb) showed strong Gd^3+^ emission at 312 nm. This emission decreased dramatically upon Tb^3+^ doping, providing clear evidence of efficient energy transfer from Gd^3+^ to Tb^3+^ ions. The energy transfer efficiency (*η_ET_*) was calculated as follows [38]:

(1) $$\eta_{\mathrm{ET}}=1\frac{\tau_{\mathrm{Gd}+\mathrm{Tb}}}{\tau_{\mathrm{Gd}}}$$

where τ_Gd_ and τ_Gd+Tb_ represent the Gd^3+^ lifetimes in the absence and presence of Tb^3+^, respectively. The decay profiles of the Gd^3+^ transition (^6^P_3/2_→^8^S_7/2_, λ_ex_ = 273 nm, λ_em_ = 312 nm) are shown in Figure S12f, with the resulting *η_ET_* values listed in Table S1. The relatively constant *η_ET_* values around 50% across different heat treatment conditions suggest that the energy transfer mechanism remains efficient regardless of crystallization degree. The dominant Gd^3+^→Tb^3+^ energy transfer mechanism is dipole–dipole interaction, supported by spectral overlap between Gd^3+^ emission (∼312 nm) and Tb^3+^ excitation (∼ 308 nm), and the ion distance (∼4.2 Å) based on unit cell parameter calculations. With unchanged *η_ET_*, luminescence enhancement arises from nanocrystallization, reducing Tb^3+^ non‐radiative relaxation (via ordered crystal field and uniform dispersion), consistent with our lifetime results, rather than from altered Tb^3+^ radiative probability. The PL quantum yield (PLQY) of GC610/8 reached 59.3% (Figure S13 and Table S1), a significant improvement compared to the precursor glass (29.8%), and highlights its high luminescence efficiency.

**FIGURE 2:**
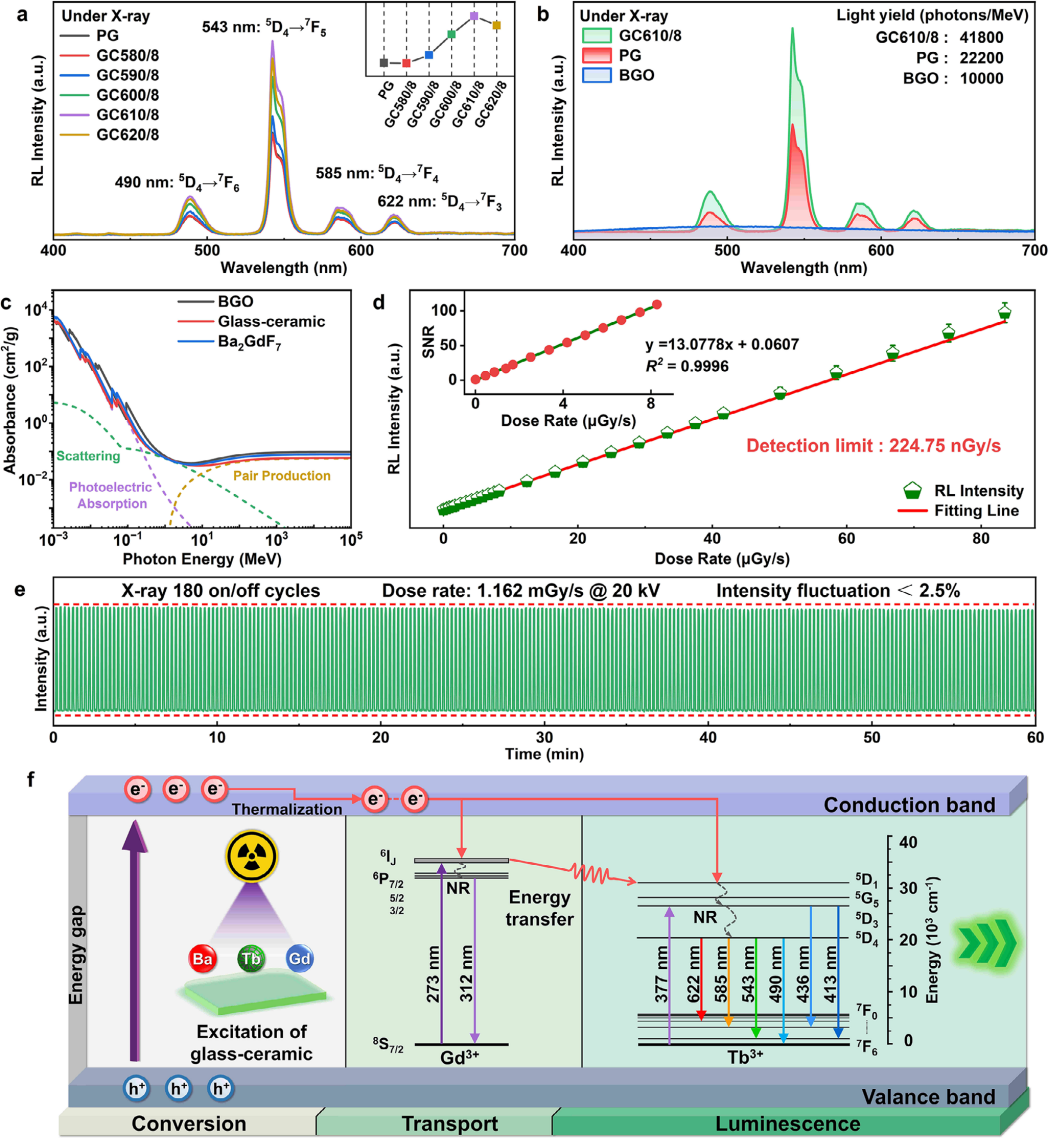
(a) Radioluminescence (RL) spectra of the precursor glass and glass‐ceramic under X‐ray irradiation (inset: intensity variation as a function of heat treatment temperature); (b) Comparison of RL spectra and light yields (LY) among the precursor glass, GC610/8 glass‐ceramic, and a commercial BGO scintillator; (c) X‐ray absorption coefficients of the glass‐ceramics, Ba_2_GdF_7_ crystal, and BGO crystal; (d) Linear relationship between radiation dose rate and luminescence intensity (inset: magnified view in the 0–10 µGy/s range); (e) Luminescence intensity of the glass‐ceramic scintillator over 180 on‐off irradiation cycles at 1.162 mGy/s (20 kV); (f) Proposed energy transfer mechanism and energy level diagram of Gd^3+^ and Tb^3+^ under X‐ray excitation.

The X‐ray excited RL spectra (Figure 2a) closely matched UV‐excited spectra in profile and intensity, confirming consistent fundamental luminescence mechanisms across excitation sources. Importantly, precursor glass and glass‐ceramics exhibited significantly higher light yields than a commercial BGO crystal scintillator (Figure 2b). The GC610/8 sample achieved a remarkable light yield (LY) of 41 800 photons/MeV, equivalent to 418% of BGO's performance. Notably, this performance is competitive with commercial single‐crystal scintillators (Table S2) [39, 40], particularly considering the relatively lower density (3.90 g/cm^3^) and effective atomic number (Z_eff_ = 47) of the glass‐ceramic compared to BGO (7.10 g/cm^3^, Z_eff_ = 74). However, high LY effectively compensates for the drawback that thicker plates are required to achieve similar X‐ray absorption, ensuring excellent detection performance while retaining the material's processing advantages.

The glass‐ceramic's X‐ray absorption coefficient (Figure 2c) was comparable to BGO [41], due to the high atomic numbers of Ba (Z = 56) and Gd (Z = 64), which offset its lower density. The scintillator also exhibited a highly linear radiation dose response (*R^2^* = 0.9996, Figure 2d) with a sensitivity of 224 nGy/s, enabling quantitative radiation measurement over a wide dynamic range. Signal stability was maintained over 60 min of continuous irradiation and 180 on‐off cycles (Figure 2e), with < 2.5% intensity attenuation, ensuring reliability in long‐term practical use. The radioluminescence mechanism involves a multi‐step energy conversion process (Figure 2f). Initially, X‐rays generate electron‐hole pairs via photoelectric and Compton effects [42, 43]; hot carriers then thermalize, transfer energy to the matrix, and excite Gd^3+^ and Tb^3+^ via resonant energy transfer. The efficient Gd^3+^→Tb^3+^ cross‐relaxation further populates Tb^3+^ excited states, resulting in radiative relaxation and characteristic visible emission via ^5^D_4_→^7^F_J_ (J = 6, 5, 4, 3) transitions. This cascade enables efficient high‐energy X‐ray‐to‐fluorescence conversion with minimal energy loss, accounting for the material's high light yield.

### 2.5 Thermal Stability of Glass‐Ceramic Scintillators

**FIGURE 3:**
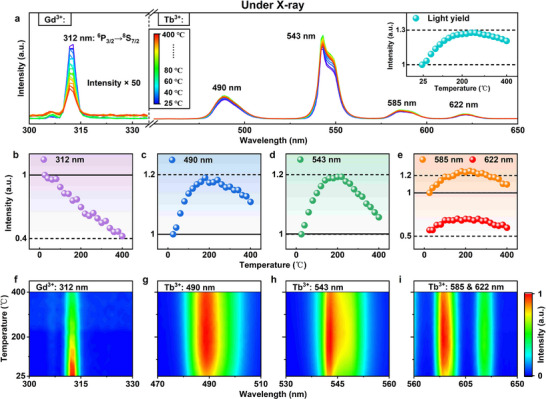
(a) XEL spectra of the glass‐ceramic scintillator at temperatures from 20 to 400 °C (inset: light yield normalized to room temperature); Normalized intensity of emission peaks at (b) 312 nm (Gd^3+^); (c) 490 nm; (d) 543 nm, and (e) 585/622 nm (Tb^3+^) as a function of temperature; (f–i) Contour plots showing the intensity variation of the corresponding emission peaks with temperature.

For practical applications, scintillator materials must maintain performance under varying thermal conditions, with high‐temperature stability critical for environments (e.g., industrial non‐destructive testing, medical diagnostics) [44, 45]. We evaluated the thermal quenching of the optimized GC610/8 glass‐ceramic scintillator via temperature‐dependent X‐ray excited luminescence (XEL) (20–400 C, Figure 3a). The Gd^3+^ emission at 312 nm decreased monotonically with increasing temperature (Figure 3b,f), typical of most luminescent materials, due to enhanced non‐radiative transitions at elevated temperatures. In contrast, the Tb^3+^ emissions (490, 543, and 622 nm) exhibited a unique non‐monotonic trend. The intensity increased up to 200 °C), reaching ∼120% of the room‐temperature value, and then gradually declined, yet remained above the initial value even at 400 °C (Figure 3c–e,g–i). This Tb^3+^ emission enhancement up to 200 °C indicates anti‐thermal‐quenching behavior, reflecting the material's high thermal stability over a wide temperature range. We attribute this phenomenon to two synergistic factors: first, the inherent thermal stability of the SiO_2_‐Al_2_O_3_‐Na_2_O glass matrix provides a stable environment that mitigates thermal degradation; second, electron traps within the glass‐ceramic network capture and store a portion of the X‐ray‐deposited energy, preventing immediate non‐radiative losses [46, 47]. Under thermal activation at elevated temperatures, the stored energy is released through thermal stimulation, partially compensating for intrinsic high‐temperature luminescence quenching. This trapping‐release balance (Figure 2f) sustains the material's superior luminescence under harsh thermal conditions, making it well‐suited for applications requiring stable operation across wide temperature ranges.

### 2.6 X‐Ray Imaging and Radiation Sensing Applications

**FIGURE 4:**
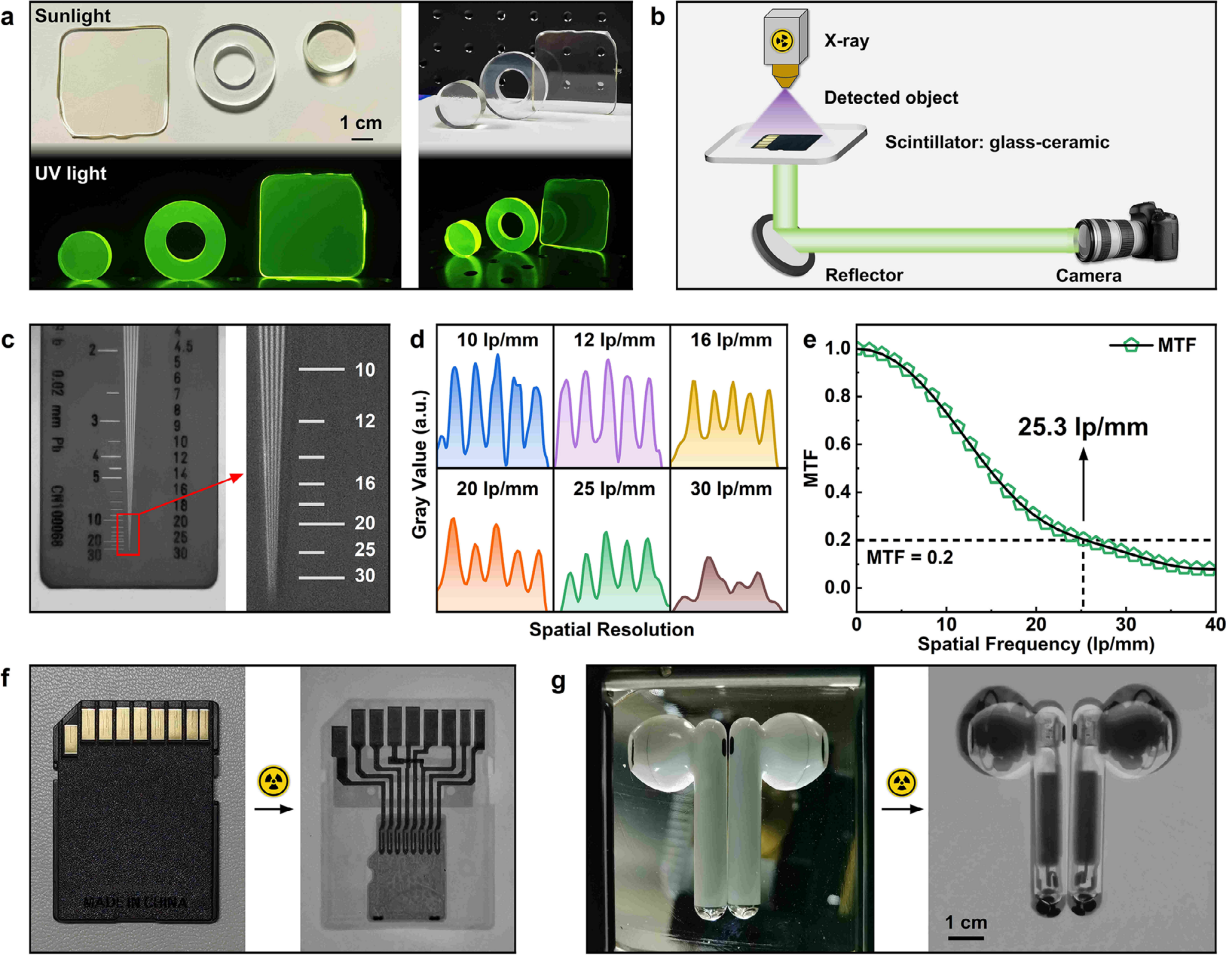
(a) Glass‐ceramic scintillator samples of different sizes and geometries under daylight (top) and UV light (bottom); (b) Schematic of the X‐ray imaging system; (c) X‐ray image of a standard line‐pair card; (d) Grayscale profiles across line‐pair groups at different spatial resolutions; (e) Modulation transfer function (MTF) as a function of spatial frequency; (f) Photographs and corresponding X‐ray image of a memory card; (g) Photographs and corresponding X‐ray image of Bluetooth earphones.

The developed glass‐ceramic scintillator exhibits excellent overall scintillation properties, with strong potential for advanced radiation imaging applications. Unlike single‐crystal scintillators, limited by size and shape customization, the excellent processability of glass enables the fabrication of scintillator elements in custom geometries and sizes to meet specific detection requirements. Figure 4a shows glass‐ceramic scintillator samples fabricated into various forms via precision cutting and polishing. Daylight and UV photographs reveal excellent optical quality and homogeneity. An X‐ray imaging system was built following standard scintillation imaging principles (Figure 4b). Spatial resolution, quantified using a standard test pattern, demonstrated clear fine‐feature resolution in the acquired images (Figure 4c). Grayscale analysis of line pairs (Figure 4d) confirmed a spatial resolution of up to 25 lp/mm, approaching that of high‐end commercial scintillators. Modulation transfer function (MTF) analysis further quantified imaging performance [48, 49, 50], showing a spatial frequency of 25.3 lp/mm at MIT = 0.2 (Figure 4e), consistent with the visual resolution results. Practical imaging was validated on complex electronic devices. X‐ray images of a memory card and Bluetooth earphones (Figure 4f,g) clearly revealed internal metallic components, circuits, and fine structural details with high contrast and spatial clarity. The Ba_2_GdF_7_: Tb^3+^ glass‐ceramic scintillator thus combines high light yield (41 800 photons/MeV; 418% of BGO), excellent spatial resolution (25.3 lp/mm), and strong thermal quenching resistance, outperforming most reported Tb^3+^‐doped glass and glass‐ceramic scintillators (Table S3). These characteristics demonstrate the material's potential for high‐resolution non‐destructive testing of precision devices and components.

**FIGURE 5:**
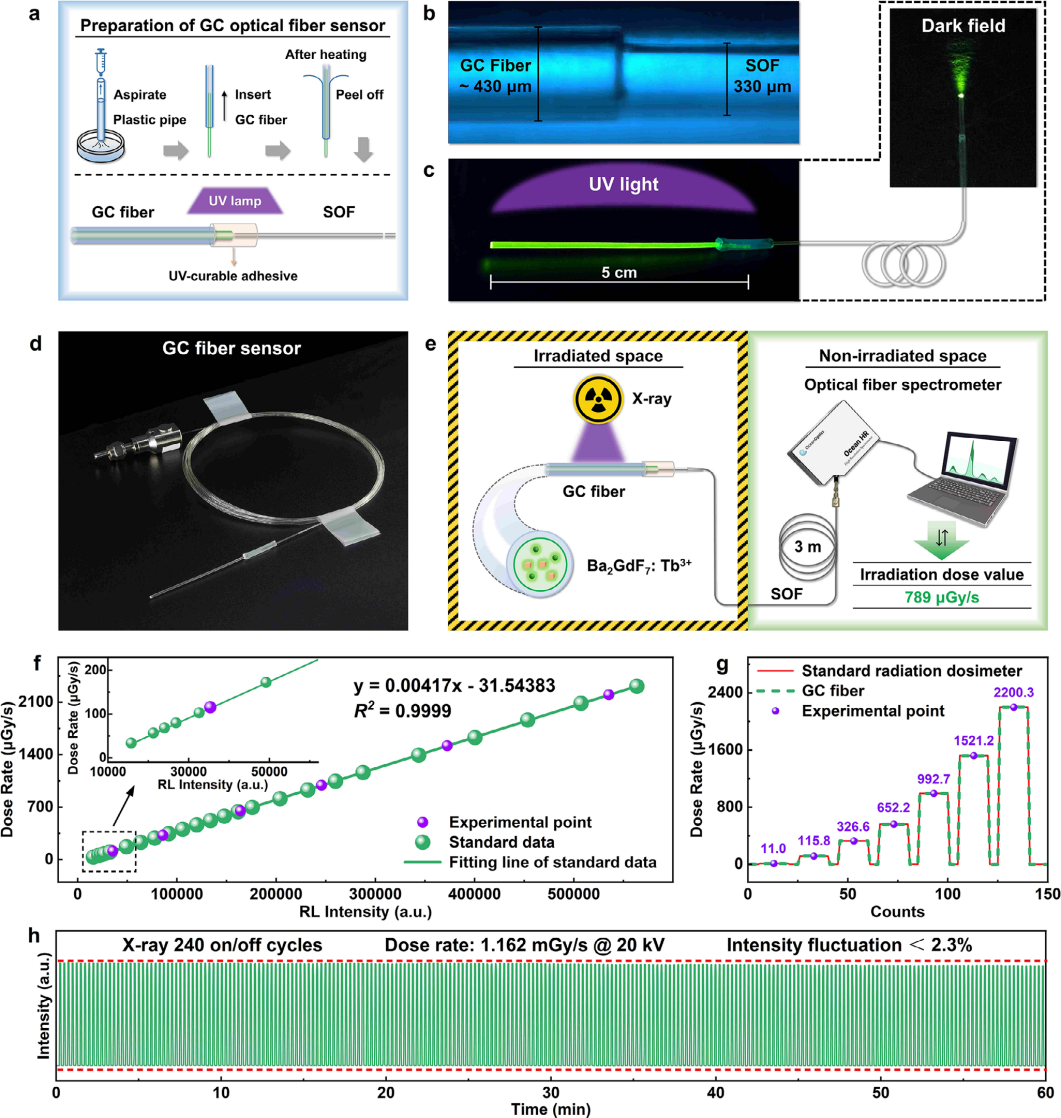
(a) Fabrication process of the glass‐ceramic fiber scintillator; (b) Micrograph of the splice between the glass‐ceramic fiber and commercial silica fiber; (c) Side‐view and end‐view photographs of the fiber under UV excitation; (d) Assembled fiber sensor module; (e) Schematic of the remote X‐ray dose detection system; (f) Linear relationship between RL intensity and X‐ray dose rate; (g) Correlation between fiber sensor readings and a commercial dosimeter; (h) Signal stability over 240 on‐off irradiation cycles at 1.162 mGy/s @20 kV.

Beyond imaging applications, the scintillator's high sensitivity, excellent dose‐response linearity, and superior processability make it attractive for radiation sensing. We developed an advanced glass‐ceramic fiber scintillator by drawing precursor glass into fibers, followed by controlled heat treatment to precipitate Ba_2_GdF_7_ nanocrystals in the fiber core. The waveguide structure enables efficient light collection and transmission, ideal for X‐ray dose monitoring in confined or hazardous environments with limited direct access. Figure 5a outlines the fabrication process, yielding a composite fiber with a core refractive index of 1.55 and a numerical aperture (NA) of 0.64, ensuring high light‐capture efficiency. A robust joint between the glass‐ceramic and commercial silica fibers was achieved via UV‐curable adhesive (Figure 5b), with bright green emission observed along the fiber under UV excitation (Figure 5c). The fully assembled fiber sensor (Figure 5d) was integrated into a remote detection system (Figure 5e), with the sensing tip isolated in an X‐ray shielding chamber and signals transmitted via a 3 m silica fiber to an external spectrometer. The system exhibited excellent dose rate linearity (*R^2^* = 0.9999, Figure 5f; Figures S14 and S15) and good agreement with commercial dosimeter readings (Figure 5g). Operational stability was maintained over 240 on‐off irradiation cycles with < 2.3% signal variation (Figure 5h), confirming practical utility for real‐time, remote radiation monitoring.

## 3 Conclusion

In this study, we developed a Tb^3+^‐doped Ba_2_GdF_7_ glass‐ceramic scintillator via a rational design integrating phase‐diagram guidance and molecular dynamics simulations. This approach established a glass system enabling efficient precipitation of fluoride crystalline nanocrystals with controlled microstructure. Optimized heat treatment yielded ∼21 nm Ba_2_GdF_7_ crystallites within the glass matrix, greatly enhancing Tb^3+^ ions’ RL efficiency while preserving excellent optical transparency. The resulting glass‐ceramic scintillator demonstrates outstanding performance, with a light yield of 41 800 photons/MeV (418%/BGO), a spatial resolution of 25.3 lp/mm, and strong thermal quenching resistance, supporting high‐resolution X‐ray imaging for non‐destructive testing. Additionally, leveraging the superior processability of the glass matrix, we fabricated flexible optical fiber scintillators and established a remote X‐ray dosimetry detection system with excellent real‐time monitoring performance and operational stability. This work introduces a high‐performance scintillator and an integrated multidisciplinary approach encompassing rational material design, precise microstructure control, and practical device engineering. Demonstrating both imaging and sensing applications highlights the versatility of the material system and offers a platform to advance radiation detection technologies in medical, industrial, and security fields.

## 4 Experimental Section

### 4.1 Preparation of Precursor Glass and Glass‐Ceramic

Precursor glasses with the composition 45SiO_2_‐15Al_2_O_3_‐10Na_2_O‐(30‐*x*)BaF_2_‐*x*GdF_3_‐*y*TbF_3_ (*x* = 8, 10, and 12 mol%, *y* = 1, 2, 4, 6, 8, 10, 12, 14, and 16 mol%) were fabricated via the melt‐quenching. High‐purity raw materials, including SiO_2_ (A.R., Aladdin), Al_2_O_3_ (A.R., Aladdin), Na_2_CO_3_ (A.R., Aladdin), BaF_2_ (A.R., Aladdin), GdF_3_ (99.99%, Aladdin), and TbF_3_ (99.99%, Aladdin), were weighed according to stoichiometric proportions and thoroughly mixed. A 30 g batch was melted in a platinum crucible at 1500 C for 1 h in air, then poured onto a preheated stainless‐steel plate (300°C) and immediately transferred to a muffle furnace for annealing at 520 C for 5 h to relieve internal stress. After cooling to room temperature, the transparent precursor glass was cut, ground, and polished into 1‐mm‐thick plates for subsequent heat treatment and characterization.

### 4.2 Characterizations

X‐ray diffraction (XRD) patterns were recorded at room temperature on a Bruker D2 Aeris diffractometer using Cu‐Kα radiation (λ = 1.5406 Å), with a scanning range of 10–80° at a rate of 10°/min. Thermal behavior of the precursor glass was analyzed via synchronous thermal analysis (STA449C) under an N_2_ atmosphere from 25 to 1000 C at heating rates of 15, 20, 25, and 30 C/min. Microstructural characterization was performed by a Talos F200X transmission electron microscopy (TEM), equipped for high‐resolution imaging (HRTEM) and energy‐dispersive X‐ray spectroscopy (EDS) elemental mapping. UV–vis transmittance spectra (250–800 nm) were measured with a Hitachi U‐3310 spectrophotometer. Photoluminescence spectra and fluorescence decays were recorded on an FLS 1000 spectrofluorometer equipped with continuous and pulsed Xenon lamps, respectively. RL spectra under X‐ray excitation were acquired using a QE Pro fiber‐optic spectrometer (Ocean Optics) coupled with a MAGPRO X‐ray source (Moxtek).

### 4.3 Fabrication of Glass‐Ceramic Optical Fibers and Sensors

Precursor glass fibers (∼ 400 µm in diameter) were drawn directly from the molten glass. The fibers subsequently underwent crystallization annealing at 610 C for 8 h to obtain glass‐ceramic bare fibers containing precipitated Ba_2_GdF_7_ nanocrystals. For cladding fabrication, PDMS base and curing agent were mixed at a 10:1 mass ratio, stirred thoroughly, and degassed under vacuum for 10–30 min. The PDMS mixture was then injected into plastic sleeves, into which the glass‐ceramic fibers were inserted. After curing at 90 C for 1 h, the sleeves were removed to form the polymer‐cladded fiber structure. Fiber end‐face coupling was achieved using UV‐curable adhesive injected into silicone tubes, with the glass‐ceramic fiber and a 330 µm commercial silica fiber inserted from opposite ends. Precise alignment under an optical microscope was performed, followed by UV curing to secure and stabilize the coupling interface.

## Conflicts of Interest

The authors declare no conflict of interest.

## Supporting information




**Supporting File**: advs74568‐sup‐0001‐SuppMat.docx.

## Data Availability

The data that support the findings of this study are available from the corresponding author upon reasonable request.
