# Supplementary material for: Tb3+‐Doped Glass‐Ceramic Scintillating Plates and Fibers for X‐Ray Imaging and Flexible Detection
Source: Adv Sci (Weinh). 2026 Feb 25;13(26):e74568. doi: 10.1002/advs.74568 (PMC13159153; doi:10.1002/advs.74568)
Supplement: Supplementary file 1 — Supporting File: advs74568‐sup‐0001‐SuppMat.docx. [file ADVS-13-e74568-s001.docx]

**Supporting Information**

**Tb^3+^-Doped Glass-Ceramic Scintillating Plates and Fibers for X-ray Imaging and Flexible Detection**

Songxuan Liu, Ping Zhang, Panpan Li, Yao Ji, Zhiguo Xia^*^, Weichao Wang^*^, Qinyuan Zhang

State Key Laboratory of Luminescent Materials and Devices, Guangdong Provincial Key Laboratory of Fiber Laser Materials and Applied Techniques, School of Physics and Optoelectronics, South China University of Technology, Guangzhou 510641, P. R. China

**Molecular dynamics (MD)** **simulation:**

Molecular dynamics (MD) simulations were performed using the LAMMPS package ^[1]^ to generate atomistic models of samples with Ba/Gd ratios of 22:8, 20:10, and 18:12. Each simulation cell contained ~ 10,000 atoms initially placed at random within a cubic box, and periodic boundary conditions were applied in all three dimensions to mitigate finite‐size effects.

The total interatomic potential consisted of long-range Coulomb interactions and short-range forces described by the Buckingham potential ^[2]^:

$V(\vec{r}_{ij})=A_{ij}\exp(-\frac{\vec{r}_{ij}}{\rho_{ij}})+\frac{q_{i}q_{j}}{4\pi\varepsilon_{0}\vec{r}_{ij}}-C_{ij}/{\vec{r}_{ij}}^{6}$ (1)

where $V(\vec{r}_{ij})$ is the potential energy between atoms *i* and *j*, $\vec{r}_{ij}$ is their separation distance, *ε*_0_ is the permittivity of free space, and *q*_i_ and *q*_j_ are the effective partial charges. To avoid unreasonable results caused by the Buckingham term at very short distances, a modified form was applied for *r*<*r*_c_:

$V'(\vec{r}_{ij})=B_{ij}/{\vec{r}_{ij}}^{n}+D_{ij}{\vec{r}_{ij}}^{2}$ (2)

where *B_ij_*, *D_ij_*, and *n_ij_* were fitted to ensure that *V*’ and its first three derivatives match the original Buckingham potential at the splice point *r*_c_. Potential parameters were taken from the Teter dataset, refined by Du and Cormack et al. ^[2]^, and validated for silicate and oxyfluoride glasses ^[3]^. Due to chemical similarity, La parameters were substituted for Gd, as commonly adopted in previous studies ^[4]^. A short-range cutoff of 8.0 Å was applied for non-Coulombic interactions, while long-range Coulombic forces were calculated using the Ewald summation method with a cutoff radius of 12 Å.

The simulation protocol began with equilibration at 4000 K for 100 ps in the canonical ensemble (NVT), followed by 30 ps in the microcanonical ensemble (NVE) to eliminate memory effects from the initial random configuration. Cooling was then carried out linearly at a rate of 5 K/ps down to 300 K under NVT conditions. At intermediate temperatures of 3000 K, 2000 K, and 1000 K, the system was equilibrated for 30 ps each in the NVE ensemble to ensure structural relaxation at progressively lower energies. After reaching 300 K, an additional 30 ps of NVE simulation was performed. During the final 30,000 steps, atomic configurations were captured every 300 steps, and structural analysis was conducted on configurations averaged over every 100 saved records.

**Reference:**

1. P. Thompson, H. M. Aktulga, R. Berger, D. S. Bolintineanu, W. M. Brown, P. S. Crozier, P. J. in 't Veld, A. Kohlmeyer, S. G. Moore, T. D. Nguyen, R. Shan, M. J. Stevens, J. Tranchida, C. Trott, S. J. Plimpton, *Comput. Phys. Commun.* 2022, **271**, 108171.
2. J. Du, A. N. Cormack, *Atomistic simulations of glasses.* **10**, 9781118939079, John Wiley and Sons. 2022.
3. a) J. Zhao, X. Xu, P. Li, X. Li, D. Chen, X. Qiao, J. Du, G. Qian, X. Fan, *J. Phys. Chem. B* 2019, **123**, 3024; b) G. Lusvardi, G. Malavasi, M. Cortada, L. Menabue, M. C. Menziani, A. Pedone, U. Segre, *J. Phys. Chem. B* 2008, **112**, 12730.
4. a) A. Wadhwa, X. Xu, Y. Huang, X. Qiao, J. Du, X. Fan, Z. Wang, G. Qian, *J. Am. Ceram. Soc.* 2024, 107, 7800; b) J. Zhao, X. Xu, K. Ren, Z. Luo, X. Qiao, J. Du, J. Qiu, X. Fan, G. Qian, *Adv. Theory Simul.* 2019, **2**, 1900062.


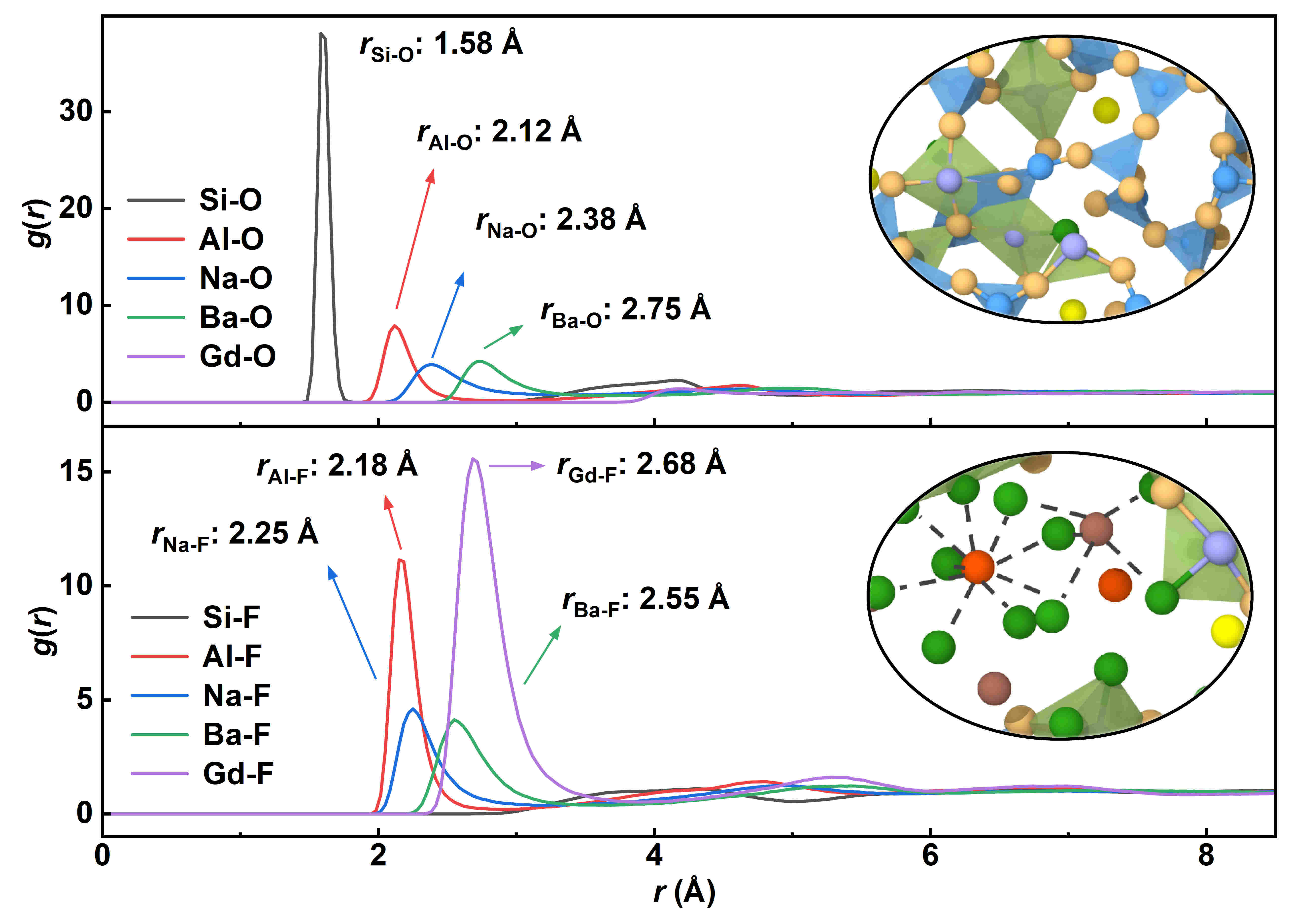


**Figure S1** Radial distribution functions of Si, Al, Na, Ba, and Gd with O and F, respectively.


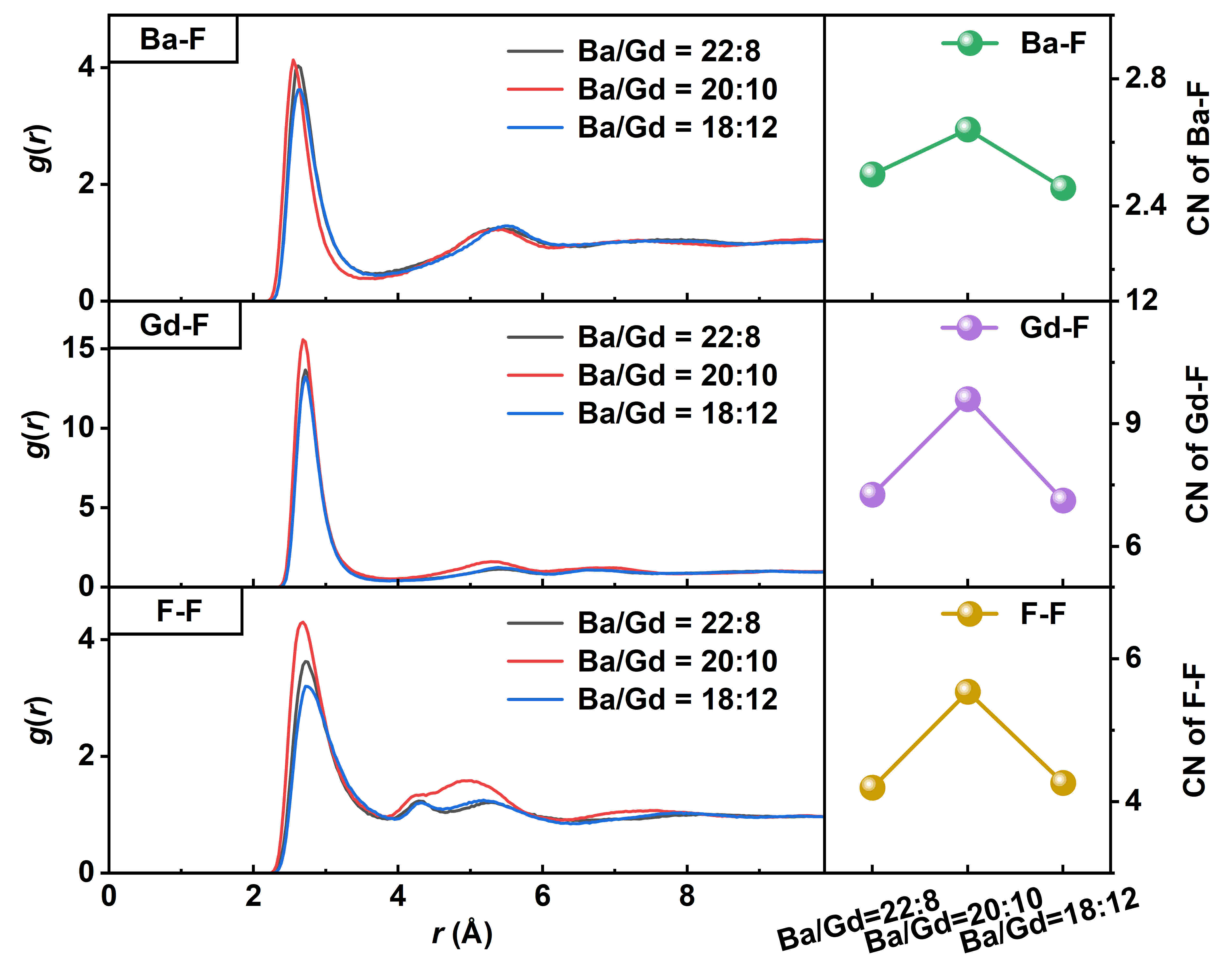


**Figure S2** Radial distribution functions and coordination numbers of Ba-F, Gd-F, and F-F pairs.


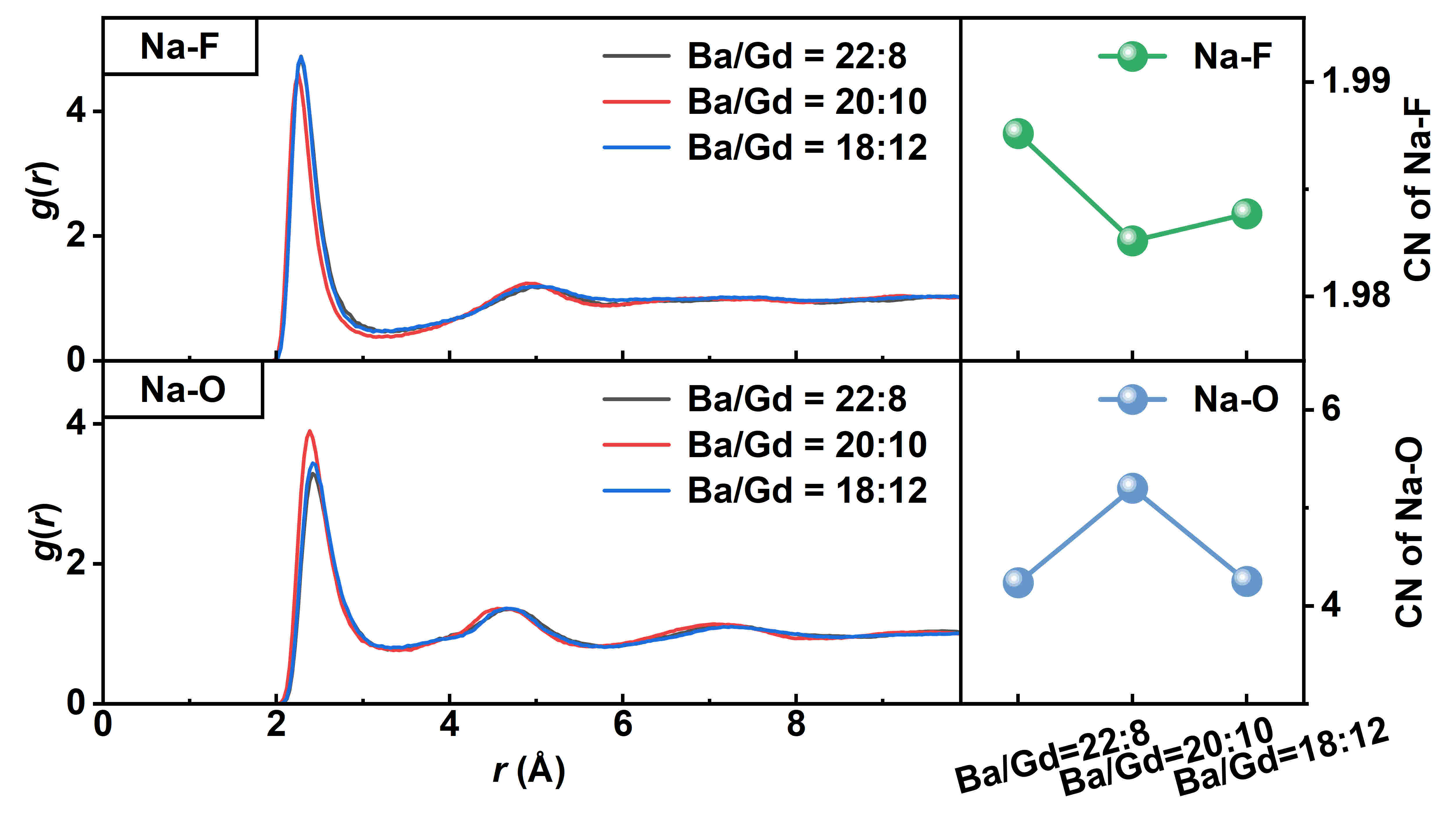


**Figure S3** Radial distribution functions and coordination numbers of Na-F and Na-O pairs.


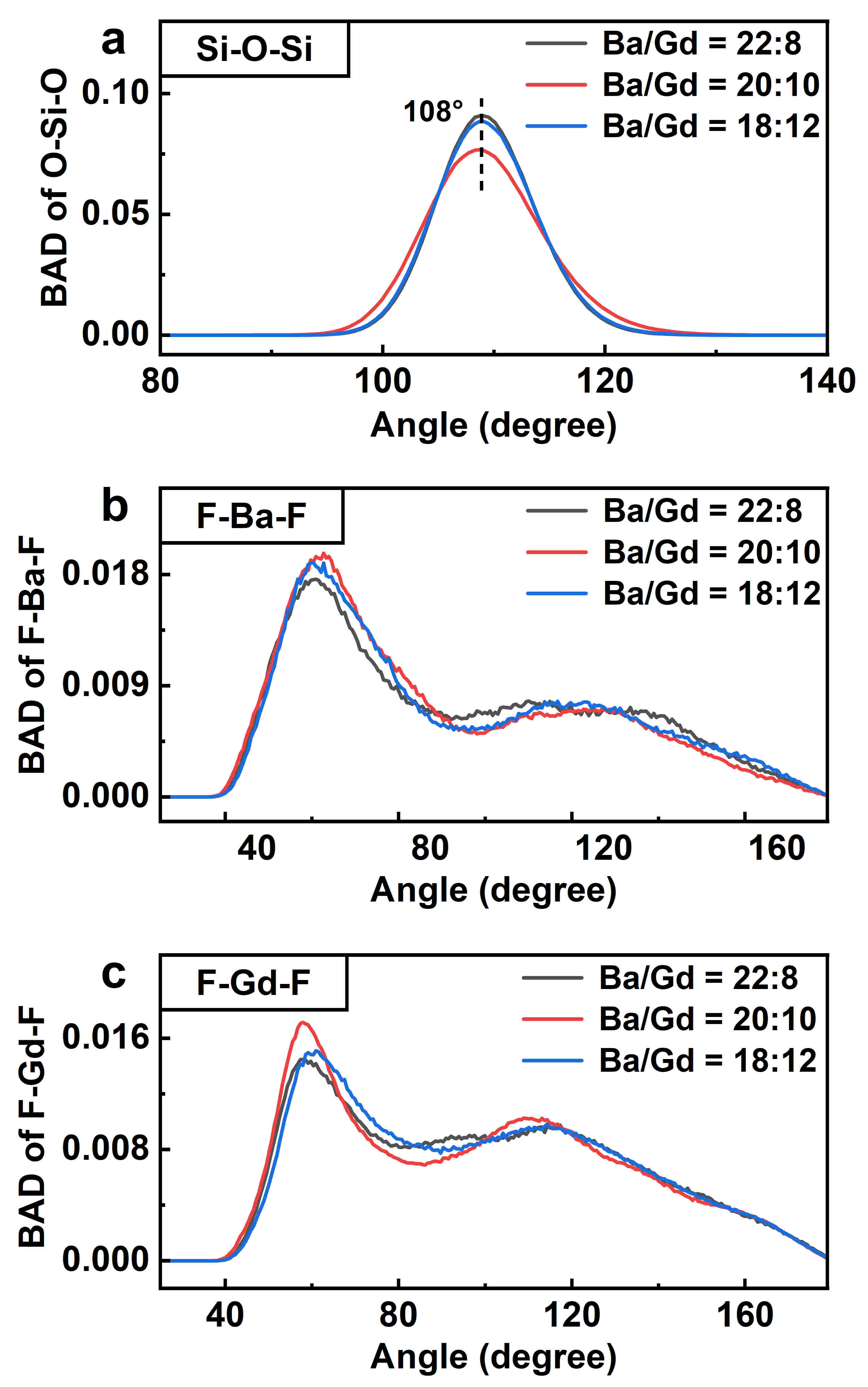


**Figure S4** Bond angle distribution of a) O-Si-O, b) F-Ba-F, and c) F-Gd-F.


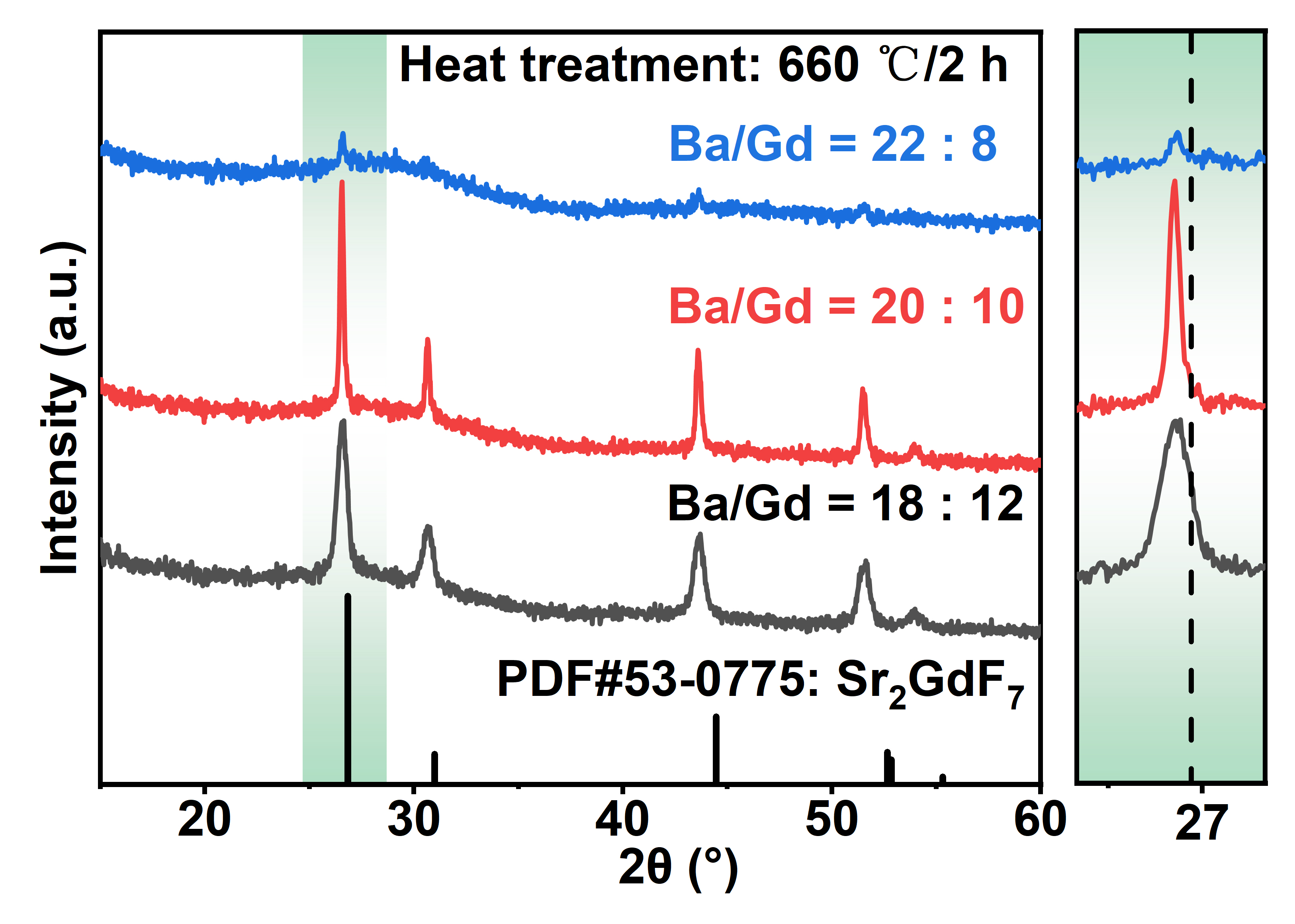


**Figure S5** XRD patterns of the glass-ceramics with different BaF_2_/GdF_3_ component ratios after heat treatment.


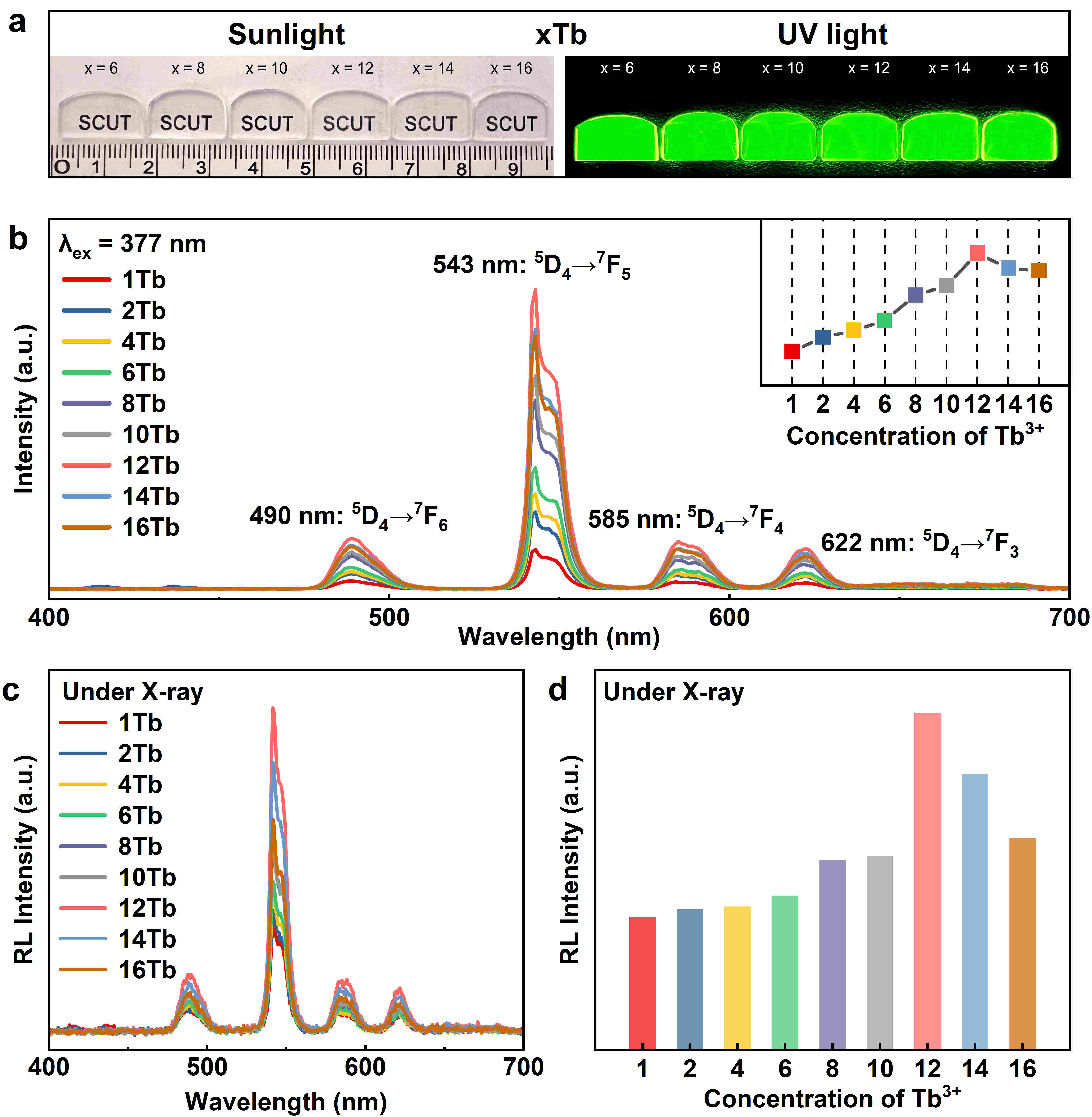


**Figure S6** a) Photographs of glasses with varying Tb^3+^ doping concentrations (*x* = 6, 8, 10, 12, 14, 16 mol%) under sunlight and UV light (λ_ex_ = 365 nm); b) Emission spectra of Tb^3+^-doped glasses under 377 nm excitation; c) RL spectra of Tb^3+^-doped glasses under X-ray irradiation; d) Variation of RL intensity with Tb^3+^ doping concentration.


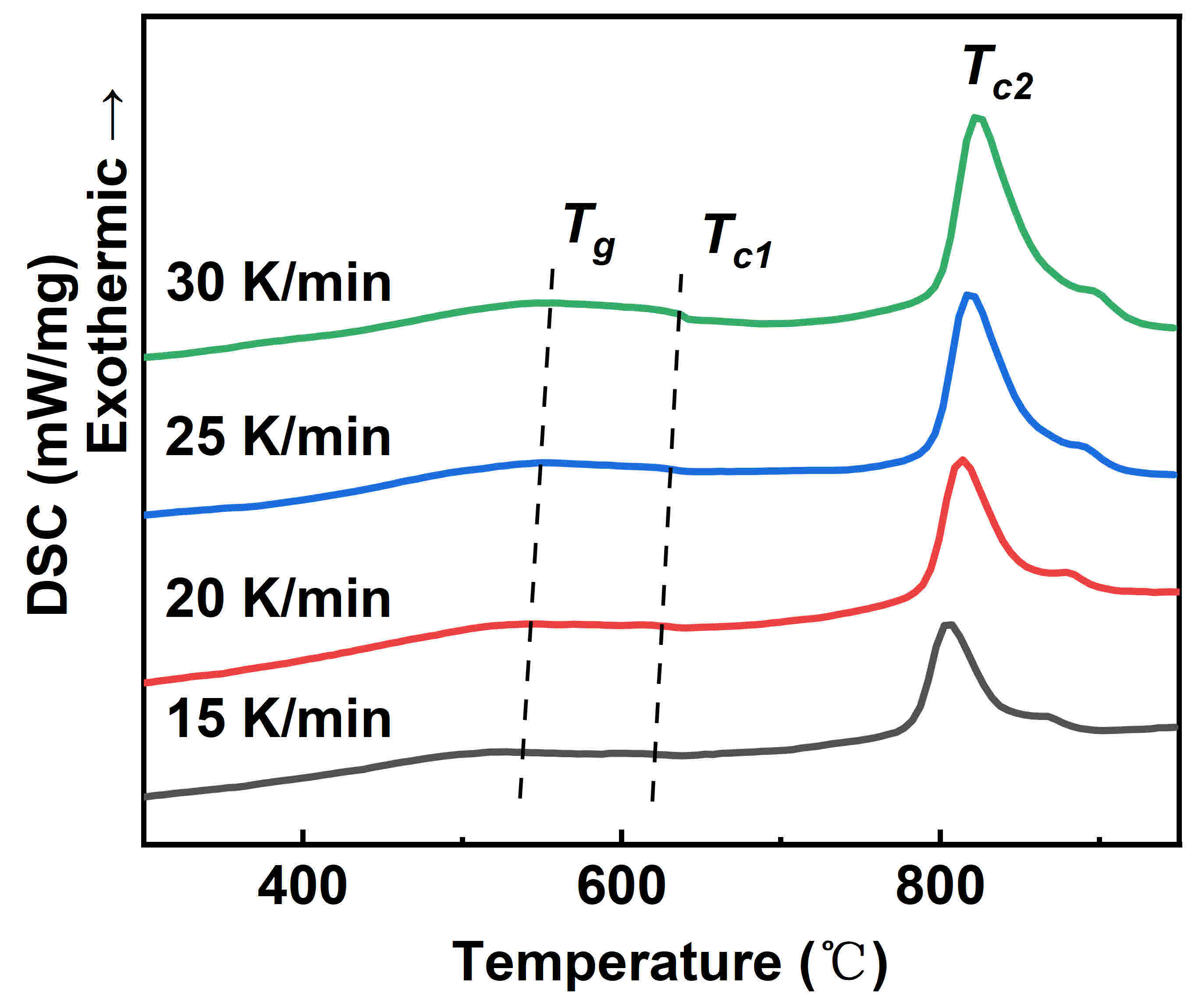


**Figure S7** DSC curves of the 12Tb precursor glass at different heating rates (15, 20, 25, and 30 K/min)

**
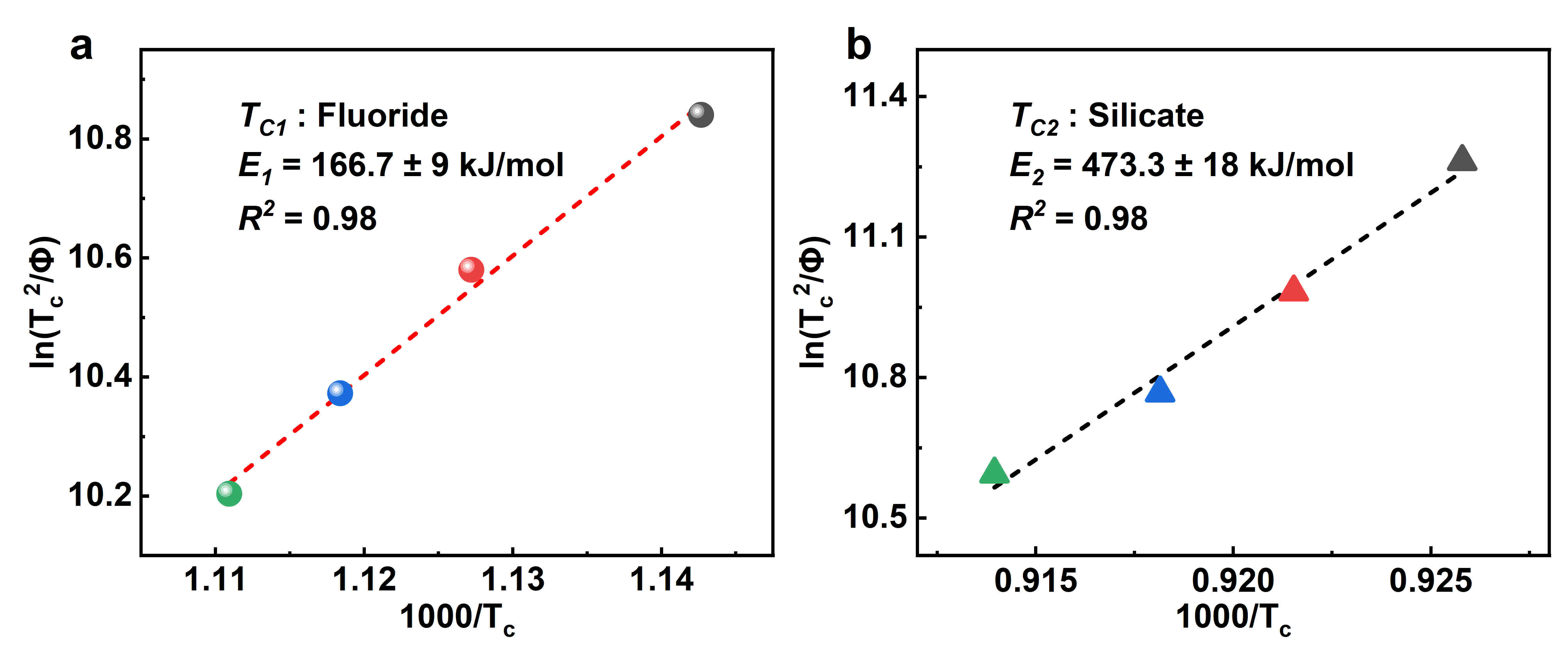
**

**Figure S8** Fitting curves of crystallization activation energy for a) fluoride and b) silicate crystalline phases.


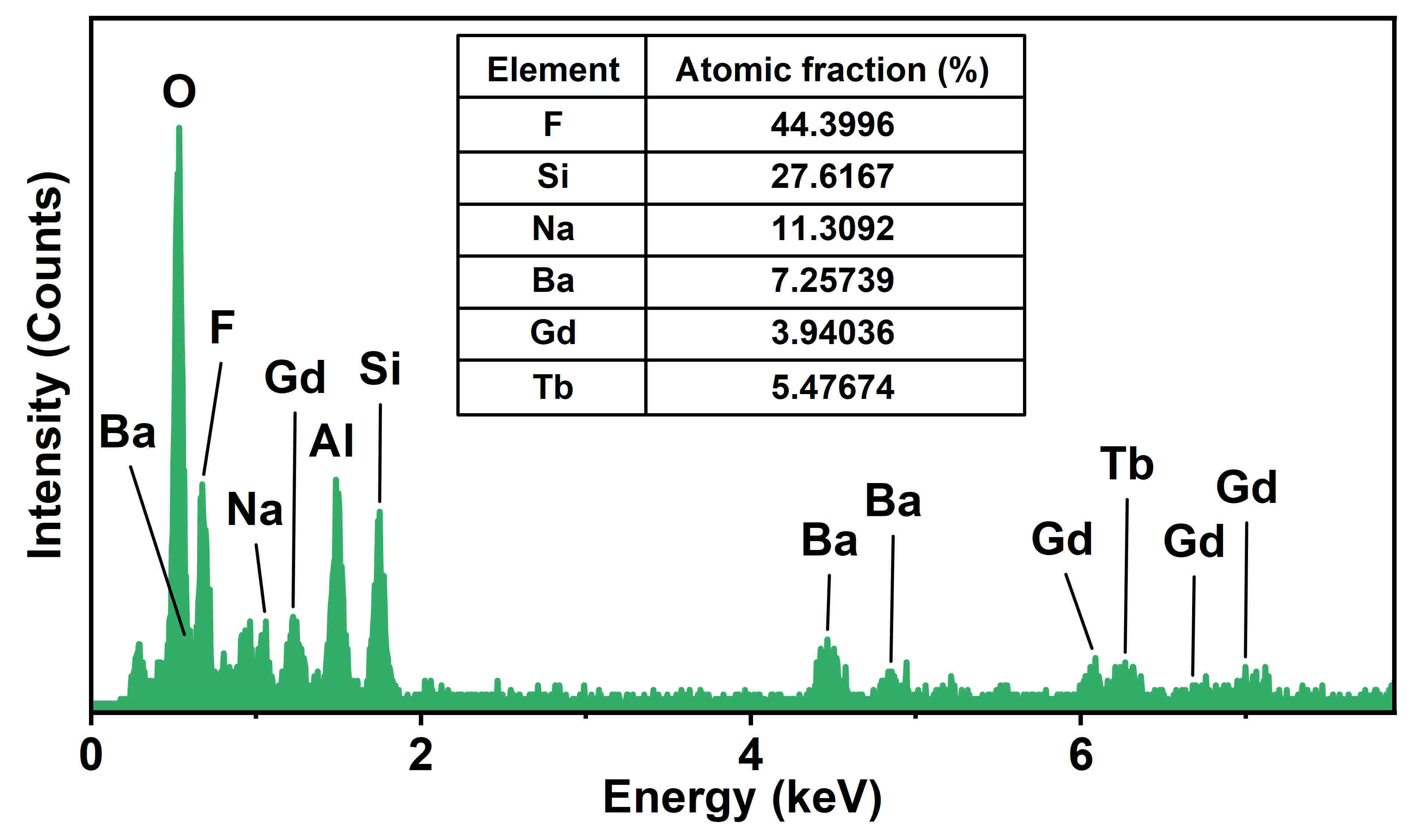


**Figure S9** Atomic fractions (%) of F, Si, Na, Ba, Gd, and Tb in the glass-ceramics determined by EDS analysis.


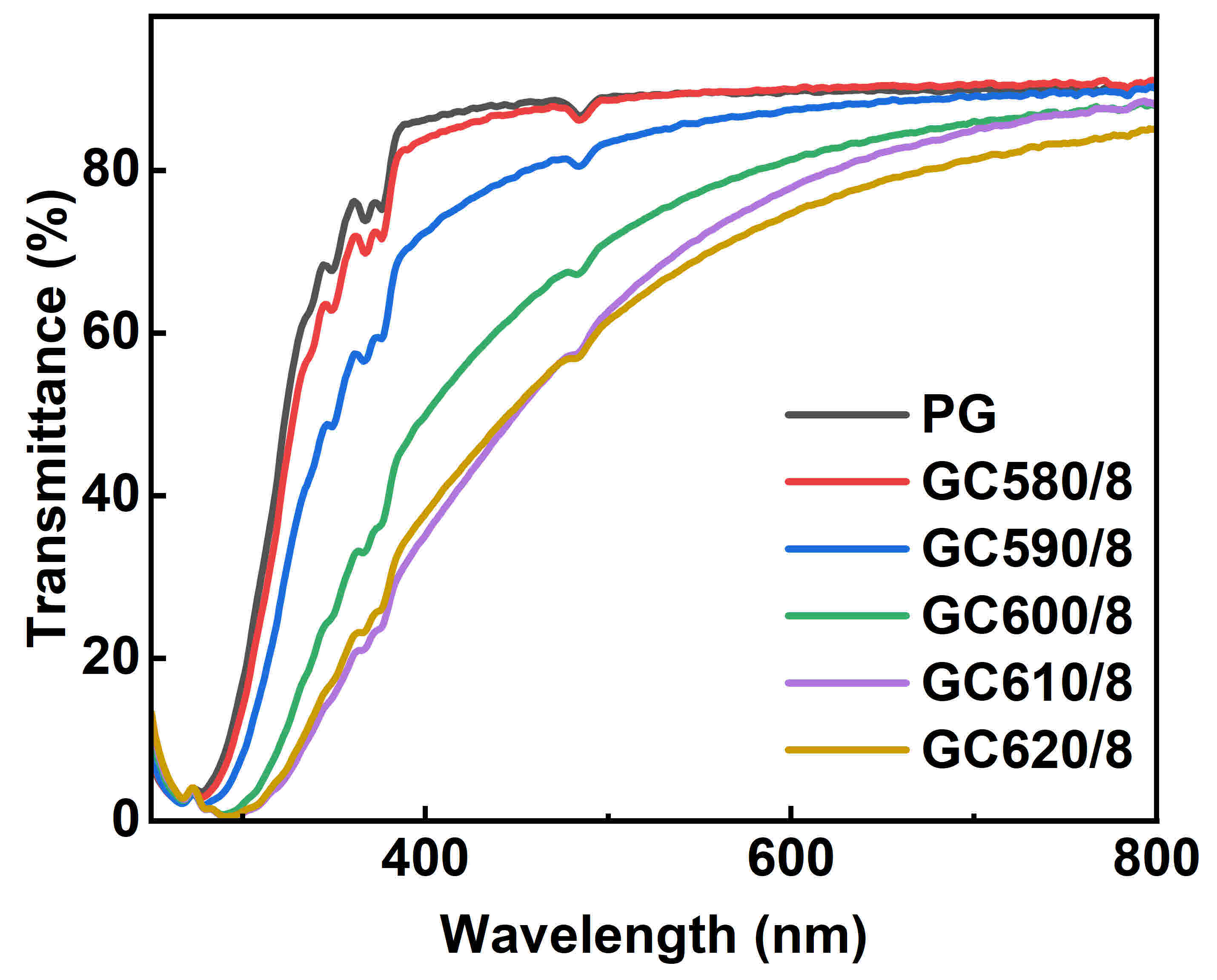


**Figure S10** Transmittance spectra of the precursor glass and glass-ceramics heat-treated at different temperatures.


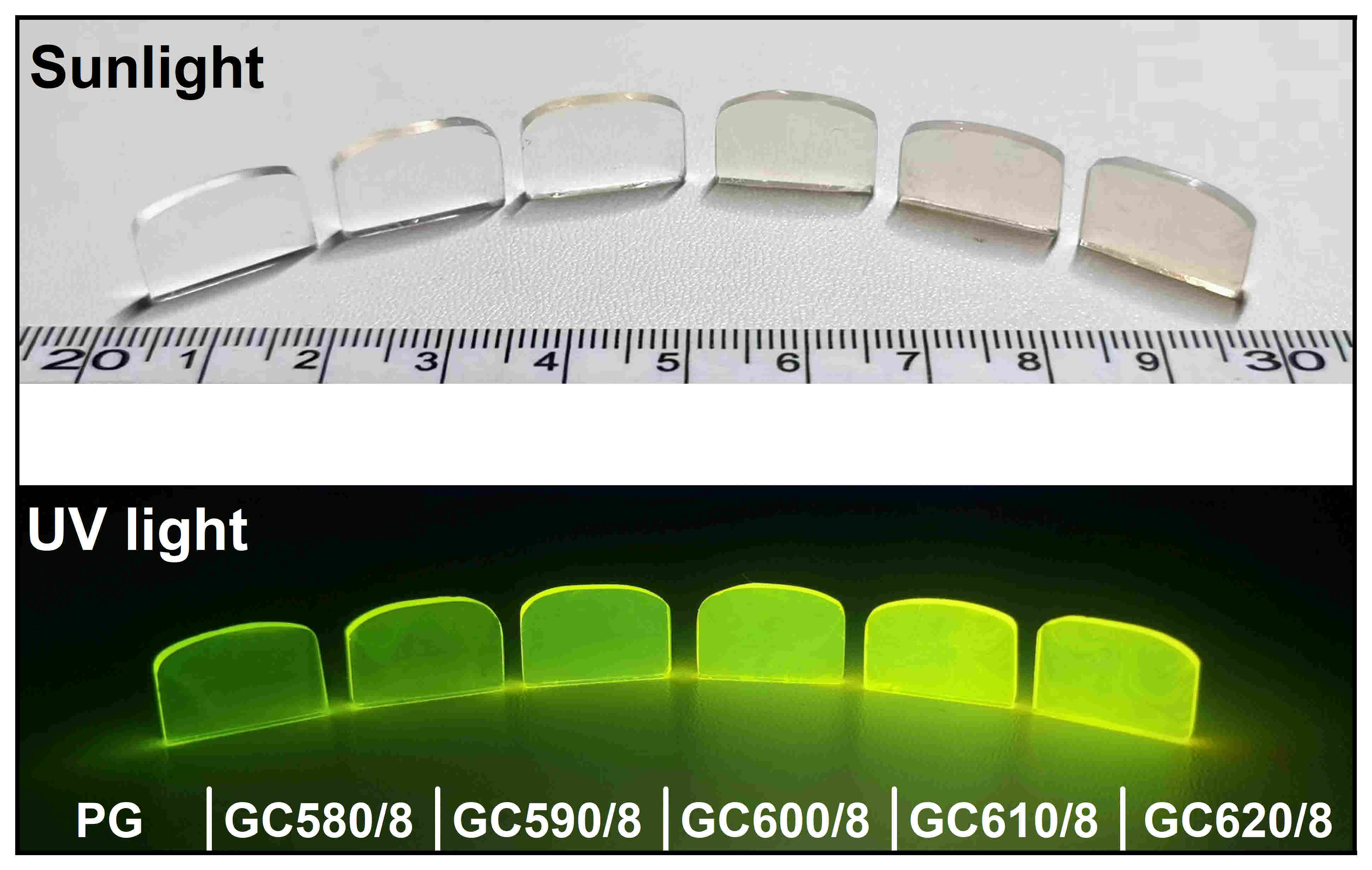


**Figure S11** Photographs of the precursor glass and glass-ceramics under sunlight (top) and UV light (bottom).


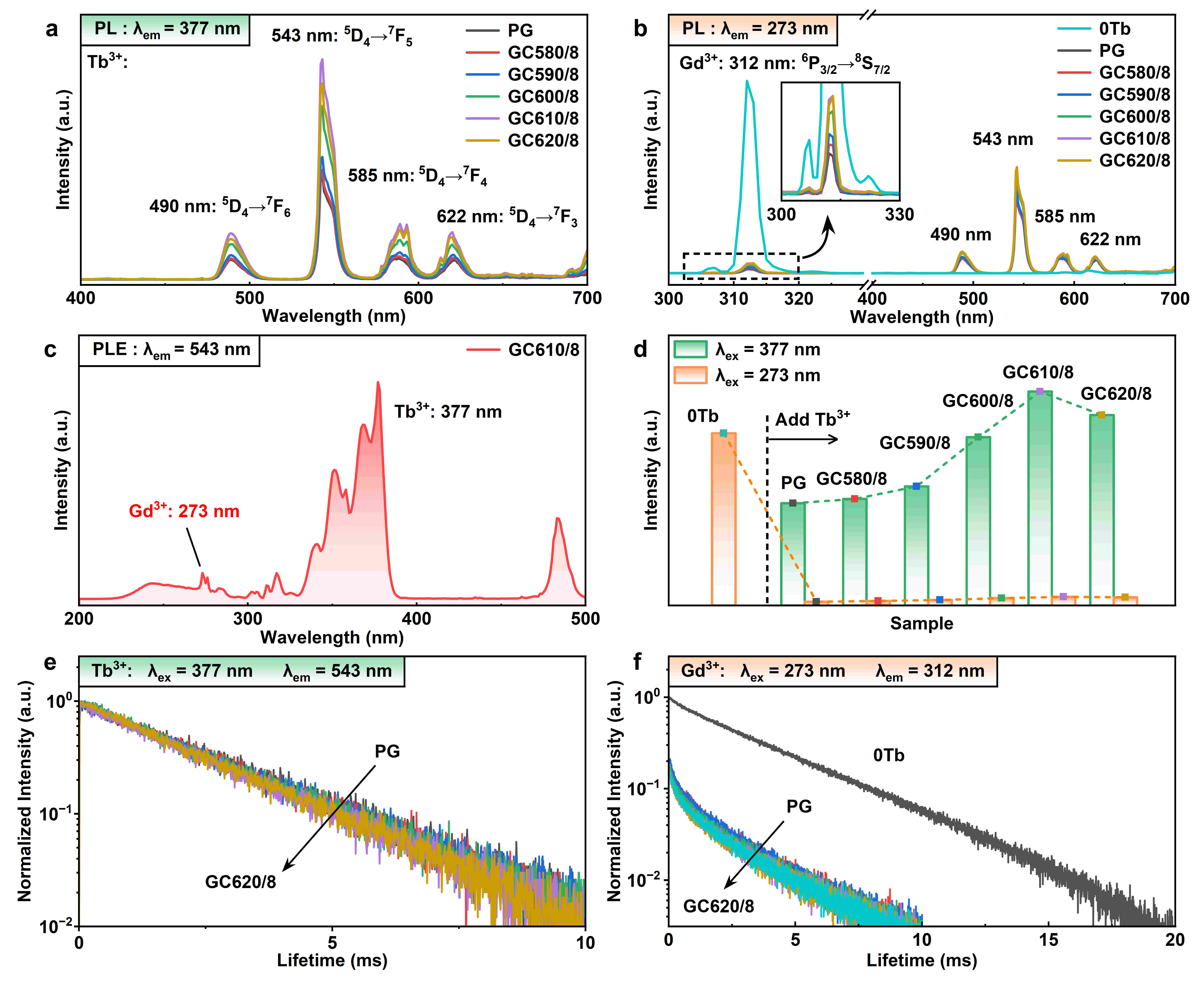


**Figure S12** a) Emission spectra of precursor glass and glass-ceramics under 377 nm excitation; b) Emission spectra of 0Tb, precursor glass, and glass-ceramics under 273 nm excitation; c) Excitation spectrum of glass-ceramics monitored at 543 nm; d) Intensity changes of 0Tb, precursor glass, and glass-ceramics under 273 nm and 377 nm excitation; e) Fluorescence decay lifetimes of Tb^3+^ ions (^5^D_4_→^7^F_5_); f) Fluorescence decay lifetimes of Gd^3+^ ions (^6^P_3/2_→^8^S_7/2_).


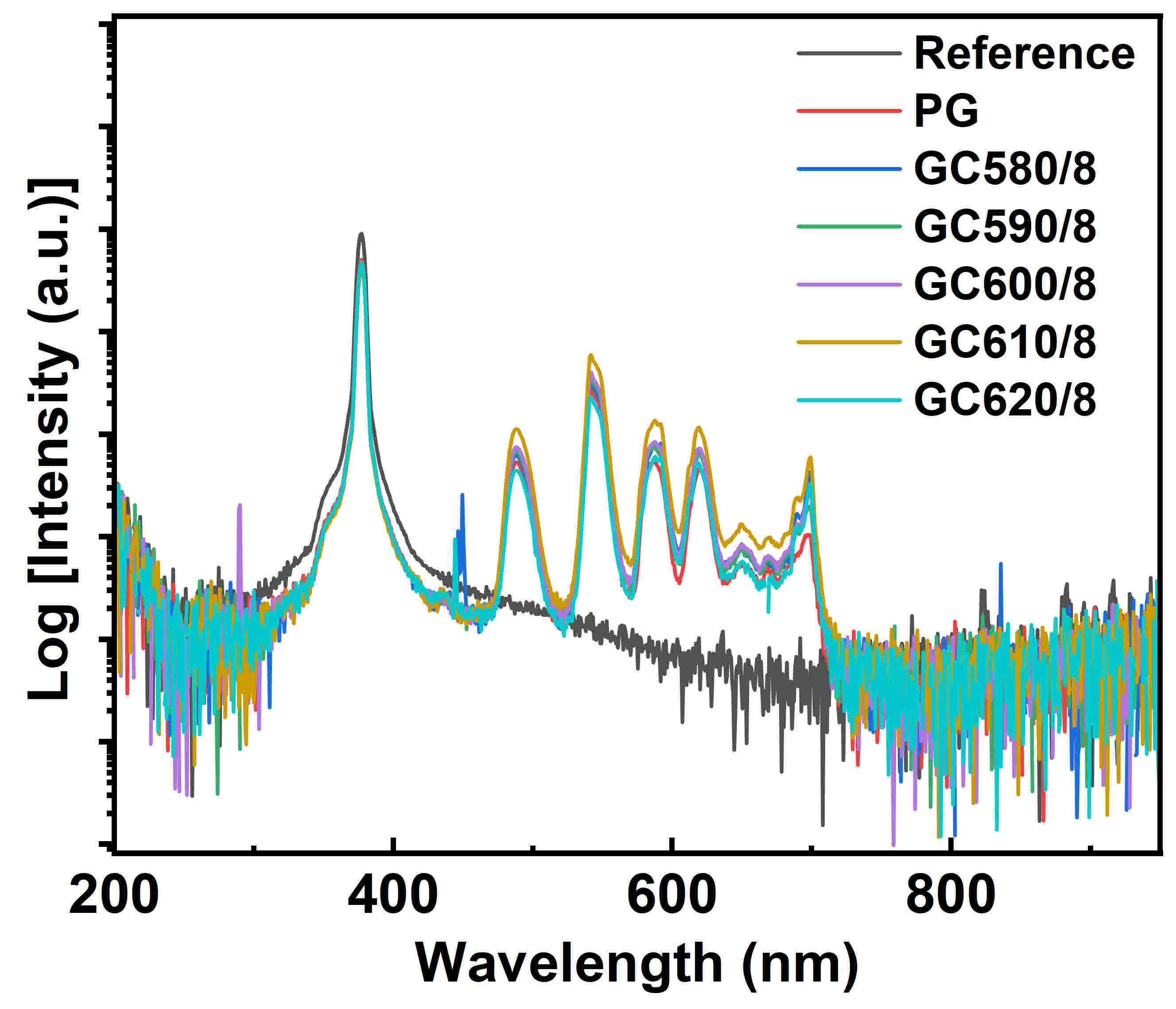


**Figure S13** Quantitative PL spectra of precursor glass, glass-ceramics, and the reference sample for absolute PL quantum yield (PLQY) determination.


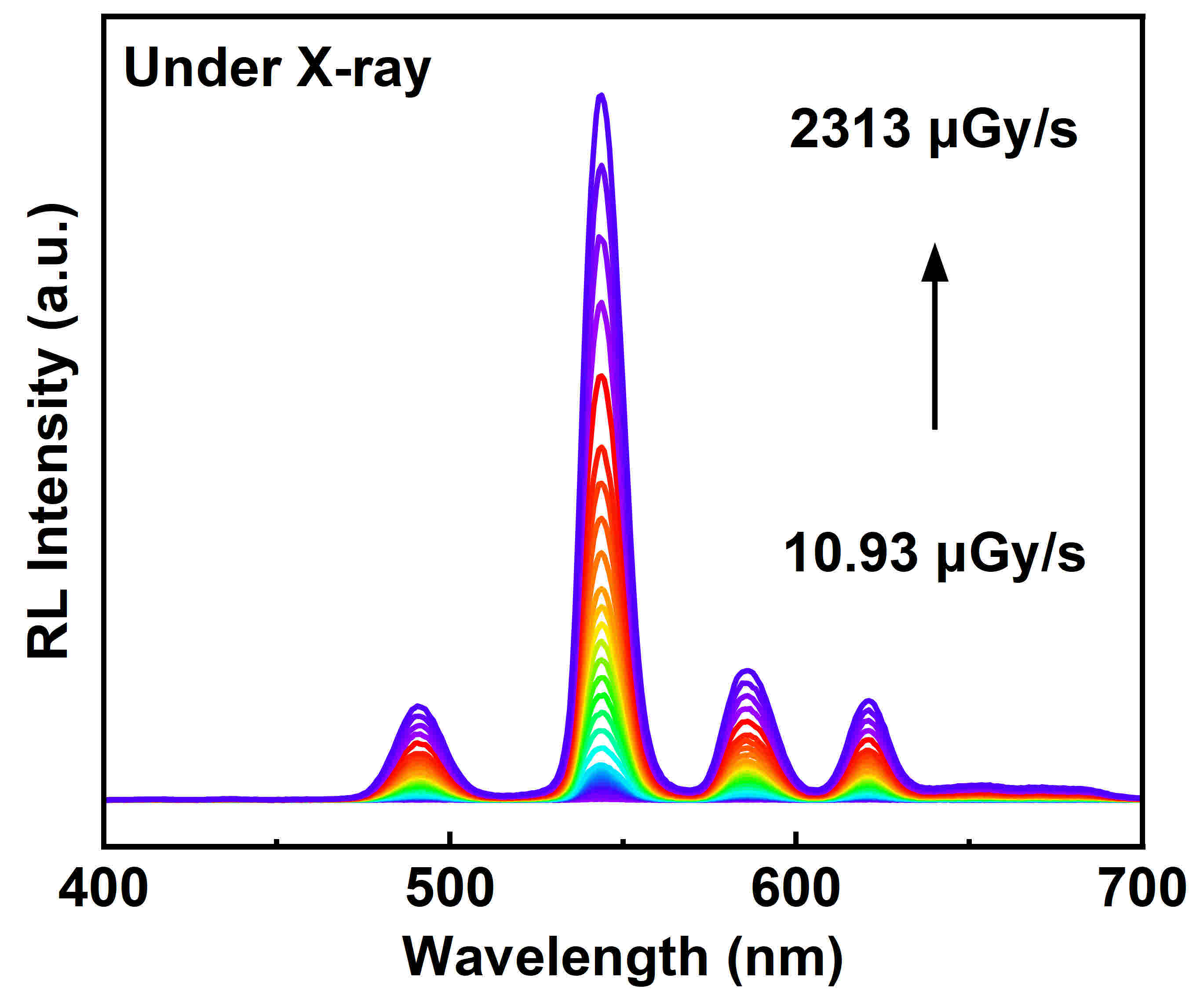


**Figure S14** Emission spectra of the glass-ceramic fiber at different radiation intensities.


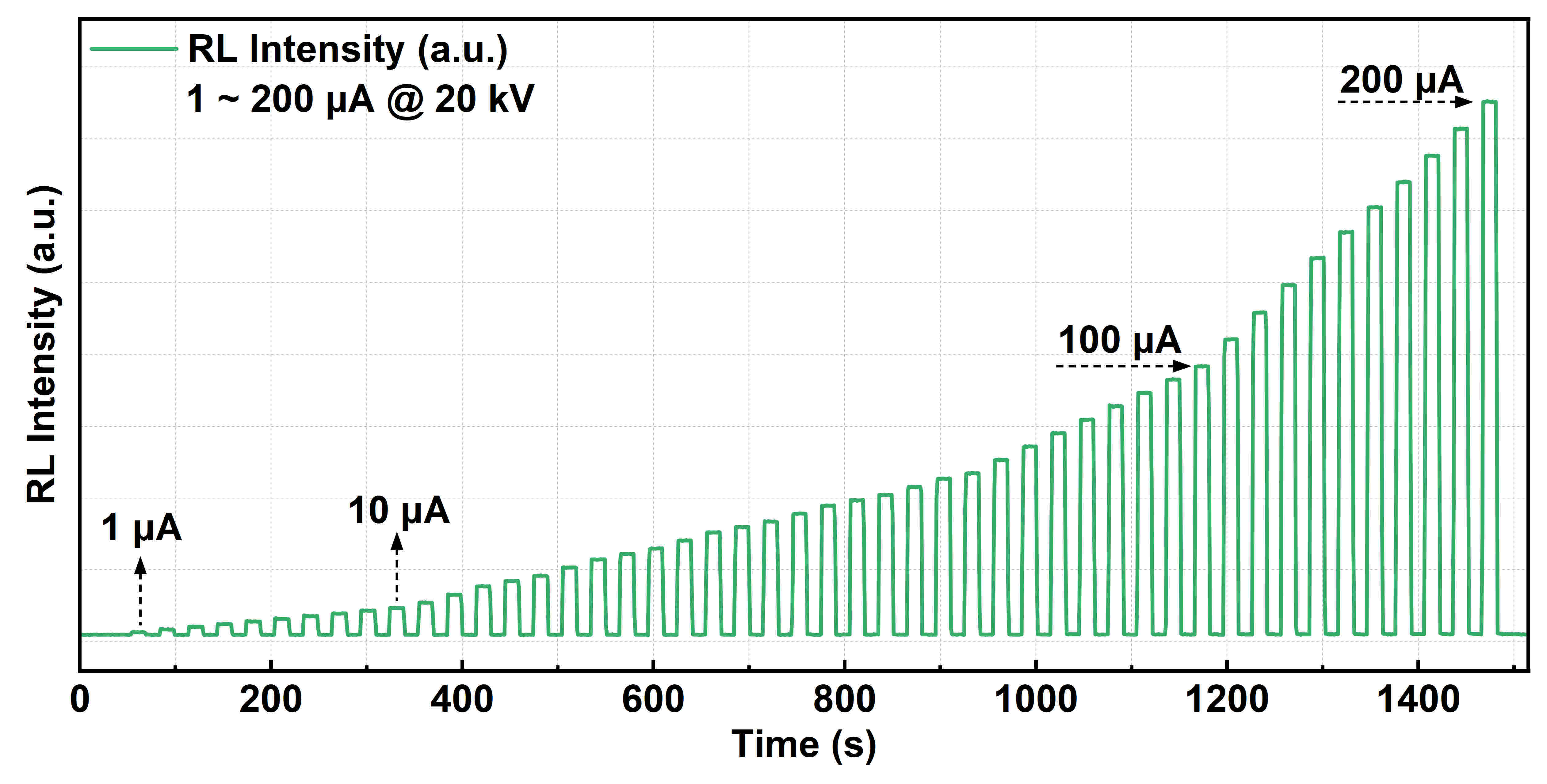


**Figure S15** Variation of emission intensity of the glass-ceramic fiber under different radiation powers.

**Table S1** Fluorescence decay lifetimes of Tb^3+^ and Gd^3+^ ions and ET efficiency from Gd^3+^ to Tb^3+^.

| **Sample** | **0Tb** | **12Tb** | | | | | |
| --- | --- | --- | --- | --- | --- | --- | --- |
|  |  | **PG** | **GC580/8** | **GC590/8** | **GC600/8** | **GC610/8** | **GC620/8** |
| τ_Tb_ (ms) | / | 2.555 | 2.361 | 2.362 | 2.279 | 2.238 | 2.211 |
| τ_Gd_ (ms) | 3.714 | 1.819 | 1.769 | 1.887 | 1.925 | 1.903 | 1.898 |
| ET efficiency (%) | / | 51.0 | 52.4 | 49.2 | 48.2 | 48.8 | 48.9 |
| PLQY (%) | / | 29.8 | 36.4 | 37.2 | 40.9 | 59.3 | 27.1 |

**Table S2** Characteristic parameters of common scintillator materials.

| **Scintillator** | **Density (g/cm^3^)** | **Effective atomic number Z_eff_** | **Dominant wavelength of luminescence (nm)** | **Decay time (ns)** | **Light yield (photons/MeV)** |
| --- | --- | --- | --- | --- | --- |
| Cs: Tl | 4.51 | 54 | 550 | 1000 | 66,000 |
| GOS: Tb | 7.34 | 59.5 | 545 | 5500 | 60,000 |
| LuAG | 6.67 | 63 | 530 | 55-65 | 25,000 |
| BGO | 7.10 | 74 | 480 | 300 | 10,000 |
| **This work** | **3.90** | **47** | **543** | **2300** | **41,800** |

**Table S3 Glass and glass-ceramics scintillator parameters reported in recent years**

| **Scintillator** | **Light yield (photons/MeV^-1^)** | **Decay time**  **(ms)** | **Detection limit**  **(nGy/s)** | **Spatial resolution (lp/mm)** | **PLQY** | **Anti-thermal properties**  **(Compared to the intensity at room temperature)** | **Ref.** |
| --- | --- | --- | --- | --- | --- | --- | --- |
| Oxyfluoride glass: Ce^3+^/Tb^3+^ | 6,726 | 2.37 | 343.9 | 16 | - | - | [1] |
| Borosilicate glass: Tb^3+^ | 10,900 | 1.88 | - | 6 | 30.8% | - | [2] |
| (Ca, Sr, Ba)_1-x_Y_x_F_2+x_: Tb^3+^ | 12,710 | 5.20 | 180 | 23.4 | 76.07% | - | [3] |
| Ba_2_LaF_7_ GC: Tb^3+^ | 13,200 | 2.33 | - | 20 | - | - | [4] |
| NaGd_2_F_7_ GC: Tb^3+^ | 14,380 | 3.74 | - | - | - | 573 K (~ 90%) | [5] |
| Borosilicate glass: Tb^3+^ | 14,700 | 2.63 | - | 20 | 53.2% | 573 K (96.7%) | [6] |
| Silicate glass: Tb^3+^ | 14,990 | 3.52 | - | - | - | 573 K (96%) | [7] |
| NaLu_2_F_7_ GC: Tb^3+^ | 15,100 | 2.27 | - | 20 | - | 573 K (~ 100%) | [8] |
| Aluminosilicate glass: Tb^3+^ | 16,300 | 2.69 | - | 20 | 50.1% | - | [9] |
| Fluorosilicate glass: Tb^3+^ | 17,100 | 2.36 | - | - | 83% | 473 K (80%) | [10] |
| Ba_0.84_Gd_0.16_F_2.16_ GC: Tb^3+^ | 19,200 | 3.50 | - | - | 64% | - | [11] |
| Sr_2_GdF_7_ GC: Tb^3+^ | 19,400 | 3.05 | - | - | 59.1% | - | [12] |
| Oxyfluoride Glass: Tb^3+^ | 20,900 | 1.96 | 1500 | 30.2 | - | 473K (86%) | [13] |
| Borosilicate glass: Tb^3+^ | 21,700 | 3.52 | - | 20 | 60.7% | - | [14] |
| Na_5_Lu_9_F_32_ GC: Tb^3+^ | 21,930 | - | - | 18.6 | - | 573 K (84%) | [15] |
| Aluminosilicate glass: Tb^3+^ | 22,400 | 2.35 | - | 20 | 51.4% | - | [16] |
| Gd-based glass: Tb^3+^ | 25,000 | 3.31 | - | 20 | 64.5% | - | [17] |
| LiLuF_4_: Tb^3+^ | 31,169 | - | 21.27 | 7.2 | - | - | [18] |
| **Ba_2_GdF_7_ GC: Tb^3+^** | **41,800** | **2.30** | **224.75** | **25.3** | **59.3** | **513 K (130%)**  **673 K (121%)** | **This work** |

**Reference:**

1. Z. Sun, X. Huang, J. Yang, S. Wang, S. Wu, *Ceram. Int.* 2023, **49**, 15500.
2. N. Intachai, S. Kothan, N. Wantana, F. Khrongchaiyapum, S. Kaewjaeng, P. Pakawanit, C. Phoovasawat, N. Chanlek, P. Kanjanaboos, N. Phuphathanaphong, H. J. Kim, J. Kaewkhao, Radiat. *Phys. Chem.* 2024, **224**, 112062.
3. Q. Liu, P. Ran, W. Chen, W. Zhang, T. Jiang, X. Qiao, Y. Yang, X. Fan, *Adv. Opt. Mater.* 2025, **13**, 2402232.
4. H. Tang, S. Liu, Z. Fang, Z. Yang, Z. Cui, H. Lv, P. Zhang, D. Wang, F. Zhao, J. Qiu, X. Yu, X. Xu, *Adv. Opt. Mater.* 2022, **10**, 2102836.
5. F. Hu, H. Gong, R. Wei, H. Guo, *J. Eur. Ceram. Soc.* 2022, **42**, 6654.
6. Y. Yuan, R. Yu, W. Dai, F. Zhang, R. Wei, H. Guo, F. Hu, *Vacuum* 2024, **230**, 113677.
7. L. Wang, F. Lu, R. Wei, T. Xiong, F. Hu, X. Li, H. Guo, X. Tian, *Ceram. Int.* 2022, **48**, 21945.
8. W. Dai, Q. Zhang, G. A. Ashraf, H. Gong, R. Wei, H. Guo, F. Hu, *Ceram. Int.* 2024, **50**, 21878.
9. S. Chen, L. Li, J. Chen, S. Xu, W. Huang, Z. Wen, T. Jiang, H. Guo, *J. Mater. Chem. C* 2023, **11**, 2389.
10. Q. Zhang, S. Zhang, R. Zheng, P. Du, J. Ding, W. Wei, *J. Non-Cryst. Solids* 2023, **608**, 122236.
11. S. Chen, W. Zhang, L. Teng, J. Chen, X. Sun, H. Guo, X. Qiao, *J. Eur. Ceram. Soc.* 2021, **4**1, 6722.
12. L. Teng, W. Zhang, W. Chen, J. Cao, X. Sun, H. Guo, *Ceram. Int.* 2020, **46**, 10718.
13. D. Zhang, S. Lin, M. Xia, Y. Rao, S. Qian, J. Ren, X. Zhang, Y. Xu, D. Chen, *Laser Photonics Rev.* 2025, n/a, 2500354.
14. W. Huang, J. Chen, Y. Li, Y. Wu, L. Li, L. Chen, H. Guo, Chin. *Opt. Lett.* 2023, **21**, 071601.
15. R. Wei, Y. Chen, L. Wang, J. Pan, X. Tian, F. Hu, H. Guo, *Adv. Opt. Mater.* 2025, **13**, 2402496.
16. L. Li, J. Chen, Z. Wen, J. Guo, Q. Wang, H. Guo, *Ceram. Int.* 2024, **50**, 757.
17. L. Li, J. Chen, X. Peng, T. Jiang, L. Lei, H. Guo, *J. Mater. Chem. C* 2023, **11**, 11664.
18. Y. Cheng, L. Lei, W. Zhu, Y. Wang, H. Guo, S. Xu, *Nano Res.* 2023, **16**, 3339.
